# Pharmacological and structural understanding of the *Trypanosoma cruzi* proteasome provides key insights for developing site-specific inhibitors

**DOI:** 10.1016/j.jbc.2024.108049

**Published:** 2024-12-09

**Authors:** Thomas C. Eadsforth, Leah S. Torrie, Paul Rowland, Emma V. Edgar, Lorna M. MacLean, Christy Paterson, David A. Robinson, Sharon M. Shepherd, John Thomas, Michael G. Thomas, David W. Gray, Vincent L.G. Postis, Manu De Rycker

**Affiliations:** 1 Wellcome Centre for Anti-Infectives Research, University of Dundee, Dundee, UK; 2 GSK, Medicines Research Centre, Stevenage, UK

**Keywords:** enzyme inhibitor, enzyme structure, molecular pharmacology, proteasome, recombinant protein expression, substrate specificity, Chagas disease, drug discovery

## Abstract

The proteasome is considered an excellent drug target for many infectious diseases as well as cancer. Challenges with robust and safe supply of proteasomes from infectious agents, lack of structural information, and complex pharmacology due to multiple active sites have hampered progress in the infectious disease space. We recombinantly expressed the proteasome of the protozoan parasite *Trypanosoma cruzi*, the causative agent of Chagas disease, and demonstrate pharmacological equivalence to the native *T. cruzi* proteasome. Active-site mutant recombinant proteasomes reveal substrate promiscuity for WT proteasomes, with important implications for assessing pharmacological responses of active-site selective inhibitors. Using these mutant proteasomes, we show that some selective parasite proteasome inhibitors only partially inhibit the chymotrypsin-like activity, including a newly developed 5-(phenoxymethyl)furan-2-carboxamide-based proteasome inhibitor. In spite of partial inhibition, these compounds remain potent inhibitors of intracellular *T. cruzi* growth. Drug-resistant mutants provide further insights in drug mode-of-inhibition. We also present the high-resolution CryoEM structures of both native and recombinantly-expressed *T. cruzi* proteasomes which reveal pharmacologically relevant differences in the ligand-binding site compared to the related *Leishmania* proteasome. Furthermore, we show that the trypanosomatid β4/β5 selectivity pocket is not present in the proteasome structures of other protozoan parasites. This work highlights the need, and provides approaches, to precisely assess proteasome substrate selectivity and pharmacology. It enables structure-guided drug discovery for this promising Chagas disease drug target, provides a new chemical starting point for drug discovery, and paves the road for development of robust proteasome drug discovery programmes for other eukaryotic infectious diseases.

Drug discovery is a resource-intensive and challenging process with high rates of attrition. Structure-guided drug discovery, integrating medicinal chemistry, structural biology, and computational approaches, enables rational design of compounds thereby both expediting progress and increasing the chances of success (1). Due to a lack of tractable and validated targets, drug discovery for parasitic diseases has been carried out mainly phenotypically. Through advances in mechanism of action studies, the targets of many of the most promising new leads for protozoan parasitic diseases have been identified. This includes EF2α (2), CRK12 (3), CPSF3 (4, 5, 6, 7, 8, 9), and the proteasome (10, 11, 12, 13). However, enabling structure-based drug discovery for these targets has proven difficult due to their complexity and challenges with purifying large amounts of these proteins from infectious agents.

The proteasome is a multisubunit protease and a key component of the ubiquitin-proteasome system which is essential for the maintenance of intracellular protein homeostasis (14). Proteasome inhibitors have great promise as antiparasitic agents (15, 16, 17), and currently two phenotypically identified *Leishmania donovani* proteasome inhibitors are progressing through clinical trials (10, 11, 12, 13). The constitutive proteasome is conserved between higher eukaryotes and protozoan parasites and comprises a 20S core particle that harbors the proteolytic activity and a 19S regulatory particle that caps the 20S particle on one or both sides (18). The 20S core particle of the proteasome consists of 28 subunits organized into four rings of seven proteins each. The two identical outer α rings limit entry to the central proteolytic barrel made up of two identical adjoining β-rings. Three of the seven β subunits harbor protease activity, β1 (caspase-like, preferential cleavage after acidic amino acids), β2 (trypsin-like, preferential cleavage C terminal to positively charged amino acids) and β5 (chymotrypsin-like, preferential cleavage after hydrophobic amino acids) (19, 20) and are expressed as proenzymes with the active site threonine only released after full assembly of the core particle through an autocatalytic mechanism (21). The generic nature of the respective substrate specificities makes developing selective substrates for each of the active sites using natural amino acids difficult (20, 22, 23). In previous work, we showed biphasic dose-response curves for many proteasome inhibitors when tested against the *Trypanosoma cruzi* proteasome using standard commercially available substrates, which may indicate turnover of the substrates by more than one of the catalytic activities of the proteasome (24).

Successful *Leishmania* proteasome inhibitors selectively target the β5 subunit. This site-preference for compounds that were identified phenotypically suggests that inhibition of the β5 site may have the most profound effect on the parasites (10, 13). A similar key dependence on β5 activity is seen in *Plasmodium* (25) as well as in higher eukaryotic organisms (26). These data indicate that inhibition of the β5 active site is sufficient to elicit cell-death. The requirement to only inhibit one of the active sites may be advantageous in terms of achieving selectivity over the human proteasome.

While several new preclinical candidates are being progressed for leishmaniasis, the pipeline for Chagas disease, caused by *T. cruzi*, another member of the Trypanosomatidae family, remains very sparse (27). Chagas disease affects approximately six to seven million people worldwide, with an estimated 1.2 million suffering from chagasic cardiomyopathy (28) and results in more than 10,000 deaths annually (29). The economic cost associated with the disease is also significant at an estimated $7.2 billion per year (30). Currently, the nitroaromatic compounds benznidazole and nifurtimox are the only drugs approved for treatment of Chagas disease. Both drugs cause severe side effects and are not consistently efficacious for the treatment of chronic Chagas disease (31), the most prevalent clinical presentation. Several compounds, all CYP51 inhibitors, have been progressed to human clinical trials but failed to show efficacy (32, 33). As a result, no new drugs have been developed for treatment of Chagas disease for more than 30 years. There is thus an urgent need to develop new drugs for this highly neglected disease and the proteasome offers a unique opportunity as a drug target.

Previous screening efforts to find new *T. cruzi* proteasome inhibitors have been limited by the amount of endogenous proteasome that can be purified from parasite cultures, by biosafety concerns that come with culturing and processing large volumes of this deadly parasite, and by a lack of understanding of partial inhibition profiles and biphasic dose-response curves (24). Recombinant expression of the proteasome would overcome the supply challenges as well as offering the opportunity to generate mutant proteasomes to address specific questions with respect to drug mechanism of inhibition. However, recombinant expression of large multisubunit protein complexes can be challenging. The recent development of multigene transfer systems such as biGBac (34) and EarlyBac (35), derived from the original MultiBac system (36), has facilitated the expression of large multisubunit complexes, including the 1.2-MDa human anaphase promoting complex/cyclosome (APC/C) and challenging membrane proteins GluN1, GluN2, and GluN3 N-methyl-d-aspartate receptors. The human and *Trichomonas vaginalis* proteasomes have also been successfully produced recombinantly using a coexpression strategy in baculovirus (37, 38). Here, we describe the recombinant expression, purification, and characterization of *T. cruzi* proteasomes and report the CryoEM structures for both the native and recombinant *T. cruzi* proteasomes. Furthermore, we generated multiple mutant recombinant proteasomes and demonstrate their value for understanding proteasome substrate specificity and drug discovery efforts.

## Results

### Expression and purification of recombinant *T. cruzi* proteasomes

The 14 subunits of the *T. cruzi* proteasome were cloned using the biGBac system for Gibson cloning and coexpressed in Sf9 insect cells (see methods). The protein subunits expressed from these constructs assembled into a complex that could be purified by nickel-affinity chromatography *via* a C-terminal His-tag on the α1 subunit (Fig. S1). Mass spectrometry of the purified complex confirmed the presence of all subunits of the *T. cruzi* proteasome (Table S1).

### Recombinant *T. cruzi* proteasome activity assessment

**Figure 1:**
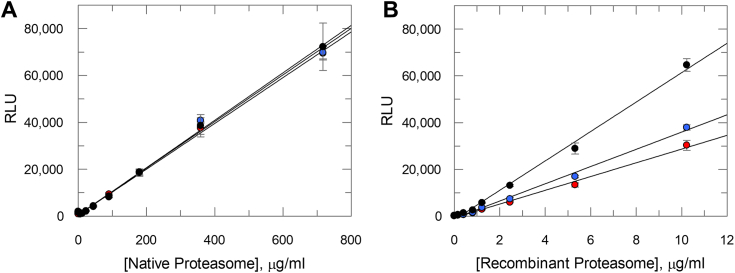
**Chymotrypsin-, trypsin-, and caspase-like activities of native and recombinant *Trypanosoma cruzi* proteasomes.** (*A*) native and (*B*) recombinant *T. cruzi* proteasomes were tested in chymotrypsin (*red circles*), trypsin (*blue circles*), and caspase (*black circles*) activity assays. Enzyme activities are plotted against *T. cruzi* proteasome concentration, with data presented as mean RLU ± SD (*n* = 3 technical replicates). *Solid lines* are linear regression of the data. In all cases the correlation coefficient > 0.99. Substrates used are suc-Leu-Leu-Val-Tyr-aminoluciferin, Z-Leu-Arg-Arg-aminoluciferin (Z is benzyloxycarbonyl), and Z-Nle-Pro-Nle-Asp-aminoluciferin for the chymotrypsin-, trypsin-, and caspase-like activities, respectively. RLU, relative luminescence unit.

Enzymatic activity of the recombinantly produced *T. cruzi* proteasome was tested alongside the previously characterized (24) natively purified proteasome from *T. cruzi*. Chymotrypsin-, trypsin-, and caspase-like activities were measured in end point assays using a luminescence-based assay (Fig. 1). The recombinant *T. cruzi* proteasome, along with the native proteasome, displayed all three enzymatic activities. For the natively purified proteasome all three activities were highly comparable, whereas for the recombinant *T. cruzi* proteasome, the caspase activity was higher than the trypsin activity, which in turn was slightly higher than the chymotrypsin activity.

**Figure 2:**
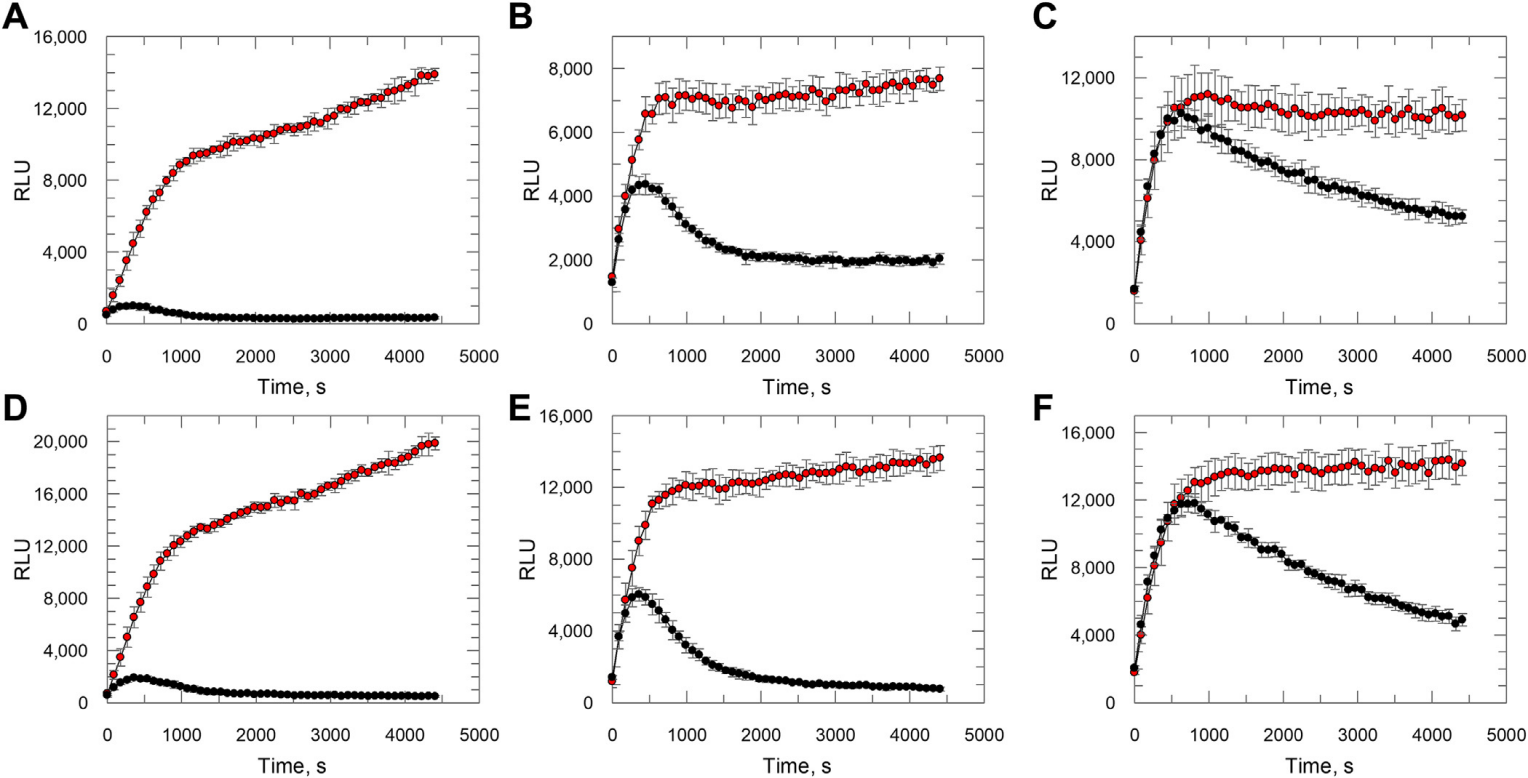
**Chymotrypsin-, trypsin-, and caspase-like activities of native and recombinant *Trypanosoma cruzi* proteasomes in the presence and absence of epoxomicin.** Chymotrypsin activity read over time for (*A*) native and (*D*) recombinant *T. cruzi* proteasomes; trypsin activity read over time for (*B*) native and (*E*) recombinant *T. cruzi* proteasomes; caspase activity read over time for (*C*) native and (*F*) recombinant *T. cruzi* proteasomes. In all graphs proteasome activity is measured in the presence (*black circles*) or absence (*red circles*) of 10 μM epoxomicin. Data are presented as mean RLU ± SD (*n* ≥ 4 technical replicates). RLU, relative luminescence unit.

Following initial confirmation that the recombinant *T. cruzi* proteasome was active, further characterization of the recombinant proteasome was achieved by measuring (side-by-side with the native proteasome) chymotrypsin-, trypsin- and caspase-like activities in the presence and absence of the irreversible proteasome inhibitor epoxomicin. Figure 2 shows enzyme activity over time, with the native *T. cruzi* proteasome activities inhibited in the presence of epoxomicin (Fig. 2, *A*–*C*). These data confirm our previously published data (24), which show that epoxomicin inhibits the different proteasomal activities to different levels, with the chymotrypsin-like activity showing the highest level of inhibition, followed by trypsin-like and caspase-like activities. Highly comparable kinetic profiles were observed with the recombinant *T. cruzi* proteasome sample in the chymotrypsin, trypsin, and caspase assays (Fig. 2, *D*–*F*), confirming that the recombinant protein is active, and that activity can be inhibited with epoxomicin.

### CryoEM structures of native and recombinant *T. cruzi* 20S proteasomes

**Figure 3:**
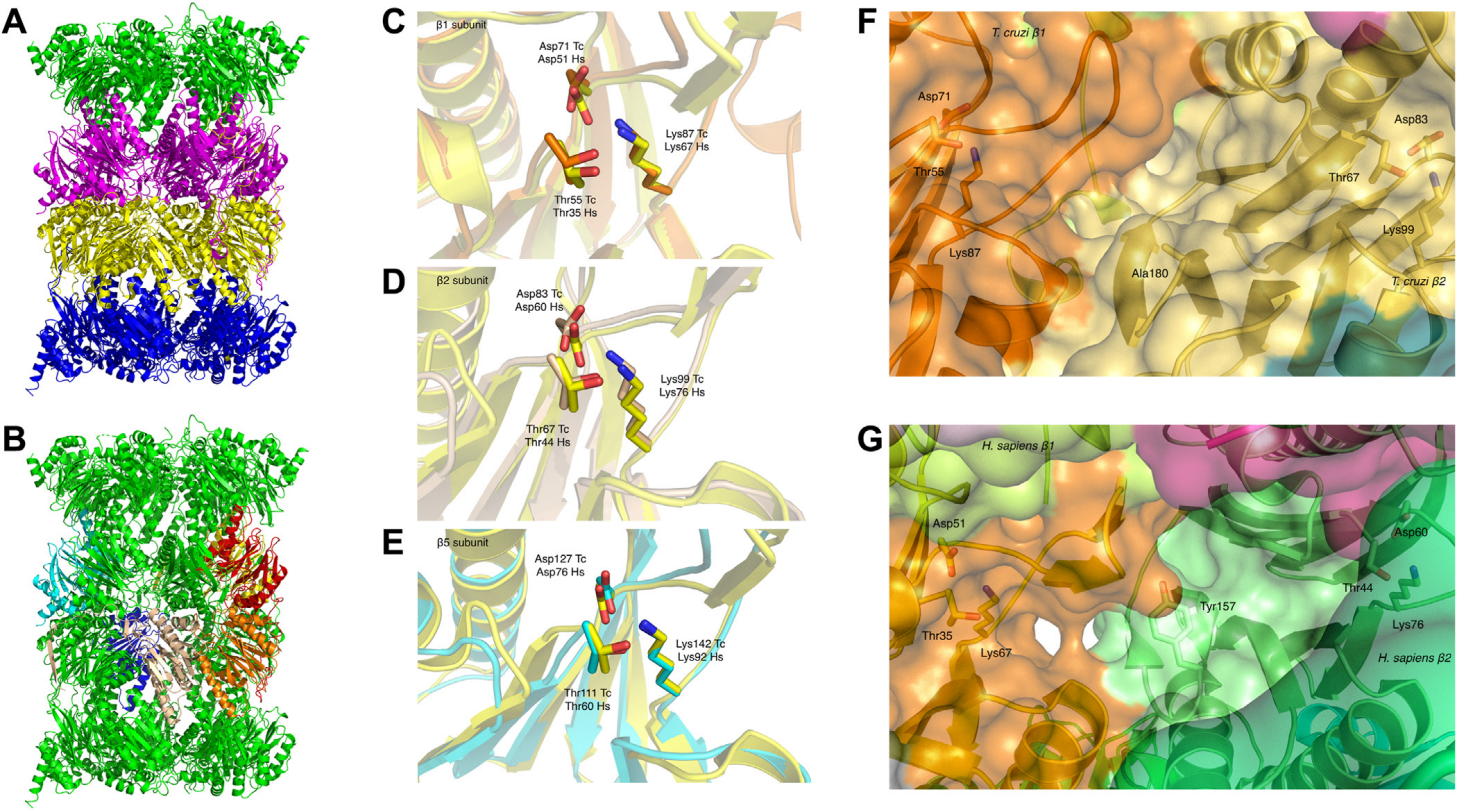
**Recombinant *Trypanosoma cruzi* apo 20S proteasome CryoEM structure.***A*, cartoon representation of *T. cruzi* 20S core recombinant proteasome (α subunits colored *green* and *blue*, β subunits *magenta* and *yellow*). Electron potential maps and refined structures for the native and recombinant *T. cruzi* proteasomes are shown in Fig. S2. *B*, orientation of catalytically active subunits β1 (*red* and *orange*), β2 (*yellow* and *wheat*), and β5 (*blue* and *cyan*) within the 20S core of the recombinant *T. cruzi* proteasome, *C*, cartoon representation of *T. cruzi* proteasome active sites; β1 (*orange*), (*D*) β2 (*wheat*) and (*E*) β5 (*cyan*) subunits with catalytic Thr 1, Asp 17, and Lys 33 highlighted compared to the orientation of the equivalent residues in the *Homo sapiens* apo 20S proteasome structure (*yellow* in all images), *F*, surface representation of the β1 and β2 active sites of the *T. cruzi* (*orange* and *wheat*) and (*G*) *H. sapiens* (*orange* and *green*) apo proteasome structures. The position of β2 Tyr 157 in *H. sapiens* fills a groove between the two active sites that is not present in *T. cruzi* due to substitution for Ala 180.

The CryoEM structures for both the native and the recombinant *T. cruzi* 20S proteasomes were determined. The native proteasome structure was determined at 2.31 Å and the recombinant structure at 2.25 Å. While the resolutions of the two electron potential maps are similar, the clarity of the recombinant protein map was considerably superior (Fig. S2), particularly for many α subunit regions, allowing accurate protein model building and confident placement of over 1000 water molecules. Figure 3, *A* and *B* show the barrel-like structure of the recombinant *T. cruzi* 20S apo proteasome, highlighting the two α1-α7 subunit rings at the ends of the barrel and the two β1-β7 rings in the center. There are only a few areas of the recombinant *T. cruzi* proteasome where the protein chains could not be modeled due to lack of density (the loops of α5 residues 125–131, α6 residues 52–58 and 201–203, and β6 residues 192–197). Where the map density was clear, the amino acid side chains were consistent with the expected protein sequences, there being no sign of any insect proteasome subunits that could potentially have been incorporated during baculovirus expression. Excepting the higher quality of the recombinant protein map there are only very limited differences apparent between the two apo structures (RMSD of 6258 aligned residues 0.85 Å). There are three cysteine residues which exhibit differences in the native and recombinant maps. In the recombinant map, the side chains of β1 Cys 174 and β6 Cys 227 show extended linear density, while β3 Cys 138 shows extended branched density, suggesting that the cysteines have been oxidized at these positions (Fig. S2*B*). The very high degree of similarity between the recombinant and native proteasome structures suggests the recombinant protein is highly suitable as a surrogate for the native protein.

### Comparison of proteasome structures

Comparison of the *T. cruzi* proteasome structure with other protozoan species (*Leishmania tarentolae* 6QM8 (liganded) and 6QM7 (apo) (13), *T. vaginalis* 8OIX (liganded) (38), *Plasmodium falciparum* 6MUW (apo) (39)), and the human proteasome (6RGQ (apo) (37)) shows that the *T. cruzi* proteasome closely resembles the *L. tarentolae* apo proteasome (RMSD of 6256 aligned residues 1.97 Å) (Table S2). The most obvious difference is that the C-terminal helices of the α3 and α4 subunits of the *T. cruzi* structure are shorter than the corresponding helices of the *L. tarentolae* structure, being less well defined in the density. The *Trichomonas, Plasmodium*, and human proteasomes are more divergent with RMSDs of 4.301 Å (6028 aligned pairs), 3.041 Å (6010 pairs), and 3.048 Å (6076 pairs) against the *T. cruzi* proteasome, respectively.

### Active sites and substrate selection

As anticipated from the fully assembled 20S core particles, the three active site subunits are all active (Fig. 1). The structure shows no density extending N terminal of the active site threonines in the map, and we thus do not observe any of the propeptides that were detected by mass spectrometry (Table S1). All three active subunits (β1–caspase-like, β2–trypsin-like, and β5–chymotrypsin-like) are defined by the conserved catalytic triad Thr 1, Asp 17, and Lys 33 (β1–Thr 55/Asp 71/Lys 87, β2–Thr 67/Asp 83/Lys 99 and β5–Thr 111/Asp 127/Lys 143) (Fig. 3, *C*–*E*). Interestingly, in *T. cruzi*, a channel connects β1 and β2 active sites, which is absent in the human proteasome due to the presence of a more bulky Tyr 157 instead of Ala 180 in *T. cruzi* in the β2 subunit (Fig. 3, *F* and *G*). The *L. tarentolae* and *T. vaginalis* proteasomes also have Ala in the equivalent position, whereas the *P. falciparum* proteasome is more like the human proteasome, with a His residue in the same position as Tyr 157 (Fig. S3).

The interaction of the substrate’s P1 side chain with the active site S1 specificity pocket determines the substrate specificity of the proteasome activities. Residue 45 at the base of the S1 pocket mainly dictates cleavage specificity, at the carboxy terminus of acidic residues for β1 (Arg 45), basic/neutral residues for β2 (Met 45), and bulky hydrophobic residues in β5 (Gly 45) (40). Overall, the S1 pocket residues responsible for P1 specificity (41) are highly similar between the *T. cruzi* and *Homo sapiens* proteasomes (Fig. S3). For β1, most residues are identical (Thr 20, Thr 35, Arg 45, Ala 49, and Gln 53), with a conservative substitution of Thr 31 for a serine in *T. cruzi*. For β2, all P1 residues are identical (Gly 45, Cys 31, and His 35), with the exception of the conservative substitution of Asp 53 to Glu in *T. cruzi* and for β5 all residues are conserved (Ala 20, Met 45, Ala 49, and Cys 52).

### β4/β5 ligand binding pocket

**Figure 4:**
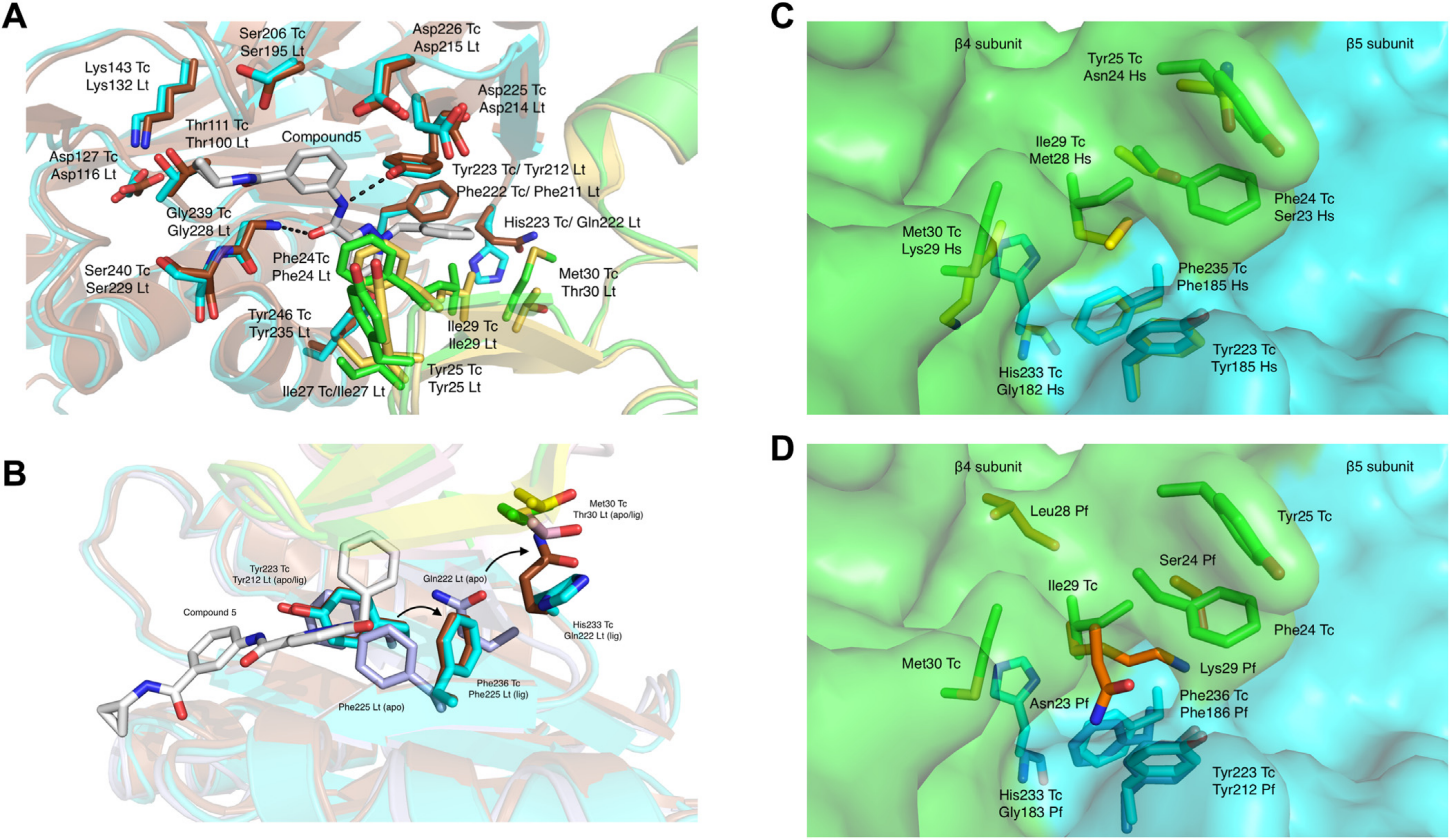
**Structural basis of selectivity.***A*, superposition of *Leishmania tarentolae* proteasome β4 (*yellow*)/β5 (*brown*) with bound “Compound 5“ (42) (*gray sticks*) onto the *Trypanosoma cruzi* proteasome β4 (*green*)/β5 (*cyan*) subunit. Residues shown as *sticks* form binding pocket within 5 Å of the superposed ligand, while the catalytic triad is also shown for reference. *B*, overlay of the recombinant *T. cruzi* apo proteasome structure (β4 (*green*)/β5 (*cyan*)) with structures of *L. tarentolae* apo (β4 (*light pink*)/β5 (*light blue*)) and complex (β4 (*brown*)/β5 (*orange*)) with “Compound 5” (*gray*) in the vicinity of the ligand binding site of the β5 subunit. *Arrows* indicate the *L. tarentolae* side chain rearrangements required to accommodate the ligand. The equivalent *T. cruzi* residues align well with the *L. tarentolae* apo side chain positions, apart from His 233 and Met 30 in *T. cruzi*, Gln 222, and Thr 30 in *L. tarentolae*. *C*, surface representation of the pocket formed between β4 (*green*) and β5 (*blue*) in *T. cruzi* with residues forming the pocket identified in kinetoplastids and humans (*yellow*) and (*D*) *Plasmodium* shown as *sticks*. Three key residue substitutions (Tyr 25 to/Asn 24 or Ser 24, Phe 24 to/Ser 23 or Asn 23, and Met 30 to/Lys 29 or Ser 29) form the basis of selectivity.

Reported kinetoplastid proteasome inhibitors selectively inhibit the parasite proteasome exploiting a hydrophobic pocket at the interface of the β4 and β5 subunits that is absent in the *H. sapiens* proteasome, as determined by CryoEM with the *L. tarentolae* proteasome (11, 13, 42). Alignment of the β5 subunits of the *T. cruzi* and *L. tarentolae* apo proteasomes shows 86.6% sequence identity (RMSD of 201 aligned residues 0.56 Å) compared to 53.2% for *T. vaginalis*, 55.7% for *P. falciparum*, and 61.2% for *H. sapiens*, while the β4 subunit is substantially more divergent (sequence identity *versus T. cruzi*: *L. tarentolae* 69.9%, *T. vaginalis* 38.5%, *P. falciparum* 34.0%, and *H. sapiens* 39.0%) (Table S2). The amino acids lining the *L. tarentolae* β4/β5 ligand binding site and selectivity pocket are highly conserved with the *T. cruzi* apo proteasome structure, with only Gln 222 in β5 and the adjacent β4 residue Thr 30 differing, the equivalent *T. cruzi* residues being His 233 and Met 30, respectively (Fig. 4*A*).

Superposition of liganded *L. tarentolae* proteasome structures show side chain rearrangements of the *L. tarentolae* apo protein β5 residues Gln 222 and Phe 225 are needed to accommodate the related ligands GSK3494245 (13) and LXE408 (11) and the pyridazinone “Compound 5” (42) in a previously identified induced fit hydrophobic pocket. However, in the case of the *T. cruzi* apo proteasome the two equivalent residues (His 233 and Phe 236, respectively) already adopt conformations comparable to those seen in the liganded *L. tarentolae* structures (Fig. 4*B*), which would make similarly large side chain rearrangements unnecessary for ligand binding. This difference is likely influenced by the larger β4 amino acid side chain of Met 30 (compared to Thr 30 in *L. tarentolae*).

Comparison of the *T. cruzi* β4 structure with human confirms the presence of a parasite-specific hydrophobic pocket that allows selective inhibition (Fig. 4*C*). Interestingly, the equivalent *T. vaginalis* and *P. falciparum* residues are more similar to the human rather than the *T. cruzi* proteasome (human: Ala 21, Ala 22, Ser 23, Asn 24, Ile 26, Met 28, and Lys 29, *T. vaginalis*: Val 21, Ser 22, Ser 23, Ser 24, Val 26, Met 28, and Ser 29, *P. falciparum*: Ser 21, Ile 22, Asn 23, Ser 24, Ile 26, and Leu 28 and Lys 29). For *P. falciparum* the polar residues Lys 29 and Asn 23 are oriented such that they occupy the space of the kinetoplastid hydrophobic pocket (Fig. 4*D*).

In contrast to the selectivity pocket at the β4/β5 interface, key β5 ligand-interacting residues are conserved across all species.

### Pharmacological validation of recombinant *T. cruzi* proteasomes

**Figure 5:**
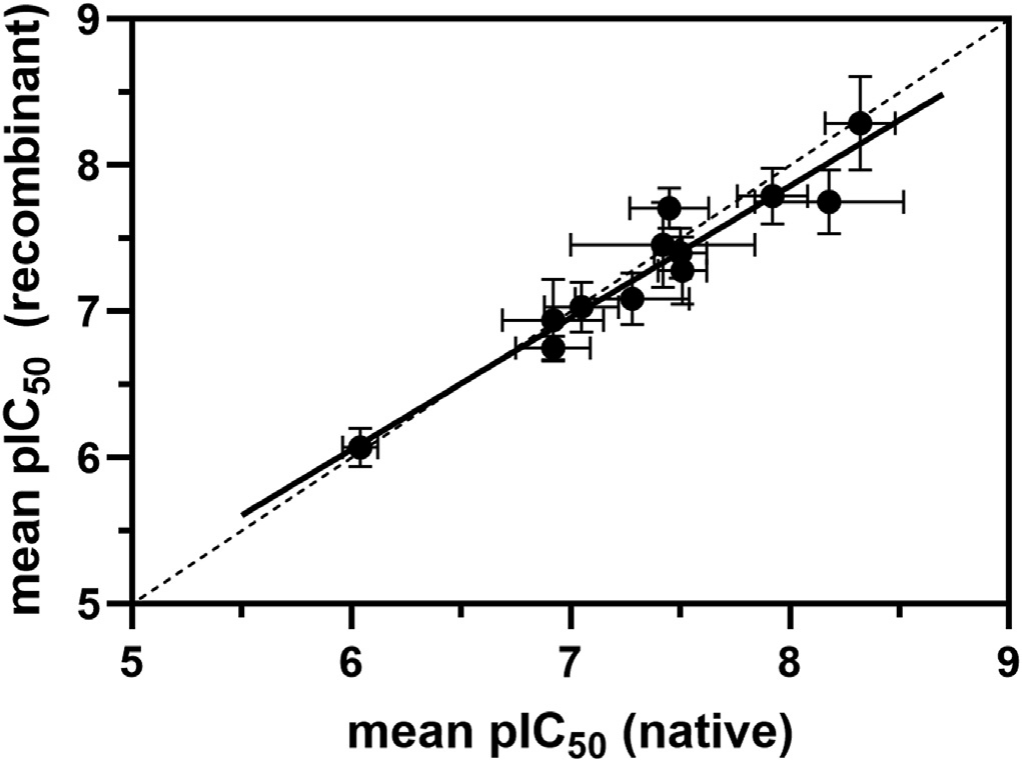
**Native *versus* recombinant *Trypanosoma cruzi* proteasome chymotrypsin assay pIC**_**50**_. Correlation between native *T. cruzi* proteasome pIC_50_ and recombinant *T. cruzi* proteasome pIC_50_ in the chymotrypsin assay for “Proteasome Reference Panel” and in-house compounds (Table S3). Data points represent mean pIC_50_ ± SD (*N* = 3 independent biological replicates). *Hashed line* is line of equipotency. *Solid line* is linear regression (R^2^ = 0.92). Mean pIC_50_ data are shown in Table S4.

To further pharmacologically validate that the recombinant *T. cruzi* proteasome is behaving in the same manner as the native *T. cruzi* proteasome, a panel of proteasome inhibitors were selected and tested in the chymotrypsin, trypsin, and caspase assays against both proteasome samples. Test compounds included a “Proteasome Reference Panel” of compounds (bortezomib, epoxomicin, ixazomib, oprozomib, MG-115, and MG-132), four examples from our previously published *Leishmania* “ES09 Series” (12, 13) (Table S3), which is also active against *T. cruzi* and inhibits the proteasome through a different mechanism than the peptide-like inhibitors in the reference panel, a compound from our pyridazinone series of *T. cruzi* proteasome inhibitors (DDD02097487) (42), and a novel *T. cruzi* proteasome inhibitor that we have developed (DDD02091966) (Table S3). Figure 5 shows that, in the chymotrypsin assay, the pIC_50_s for all compounds are very similar between the native and recombinant *T. cruzi* proteasomes (R^2^ = 0.92). Likewise, we observed comparable potencies for inhibitors of the trypsin and caspase activities between native and recombinant proteasomes (Table S4). DDD02091966 proved to be highly chymotrypsin selective, with no inhibition of the trypsin and caspase activities observed. These data demonstrate that, pharmacologically, the recombinant *T. cruzi* proteasome is behaving as the native *T. cruzi* proteasome.

### Mutagenesis of *T. cruzi* proteasome

We recombinantly produced two different mutant proteasomes; a β1/β2 inactive proteasome (Δβ1Δβ2), which only retains β5-activity and a proteasome that is resistant to inhibition with the previously reported “ES09 Series” of kinetoplastid-selective proteasome inhibitors (β4^R^) (10, 11, 12, 13, 43). For Δβ1Δβ2, the active site threonine in both the β1 (Thr 55) and β2 (Thr 67) subunits was mutated to alanine (Fig. S3). Previous work with human and archaebacterial proteasomes shows that these active site mutations still allow formation of functional proteasomes that retain catalytic activity in the nonmutated subunits (44, 45). For the production of inhibitor-resistant *T. cruzi* proteasomes (β4^R^), we generated two mutations in the β4 subunit (Ile 29 to Met and Phe 24 to Ile) based on previous resistance generation experiments and structural data of the closely related *Leishmania* proteasome (10, 13). These mutations reduce the size of the key hydrophobic binding-pocket at the β4/β5 interface and are expected to prevent binding of the “ES09 Series” inhibitors.

### Mutant *T. cruzi* proteasomes activity assessment

**Figure 6:**
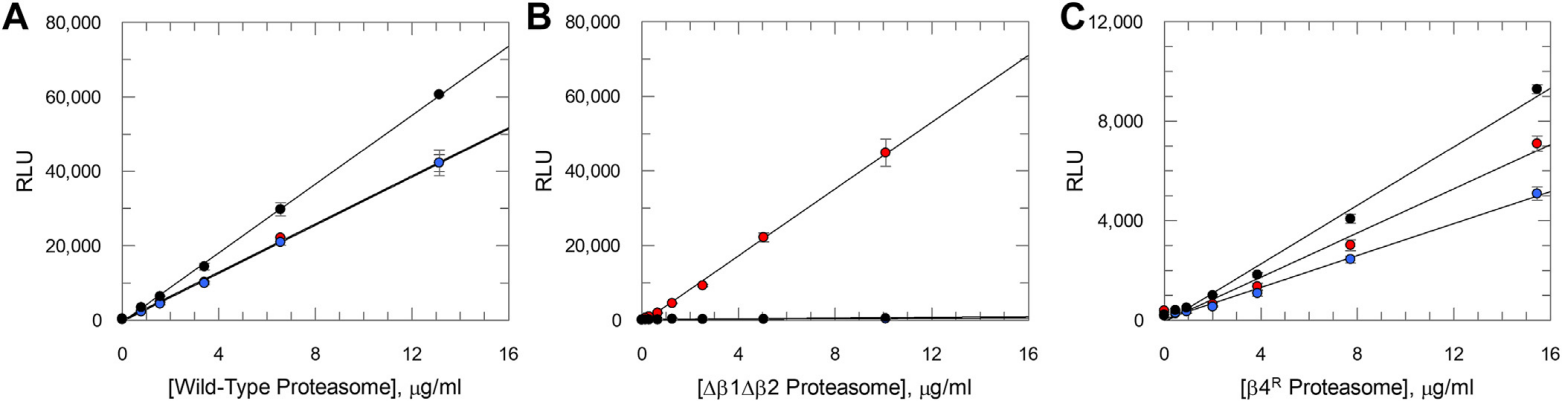
**Chymotrypsin-, trypsin-, and caspase-like activities of mutant *Trypanosoma cruzi* proteasomes.***A*, recombinant WT, (*B*) recombinant Δβ1Δβ2 mutant, and (*C*) recombinant β4^R^ mutant *T. cruzi* proteasomes were tested in chymotrypsin (*red circles*), trypsin (*blue circles*), and caspase (*black circles*) activity assays. Enzyme activities are plotted against *T. cruzi* proteasome concentration, with data presented as mean RLU ± SD (*n* = 3 technical replicates). *Solid lines* are linear regression of the data. In all cases where activity is observed correlation coefficient > 0.98. RLU, relative luminescence unit.

Enzymatic activities of β4^R^ and Δβ1Δβ2 mutant *T. cruzi* proteasomes were tested alongside the WT recombinant *T. cruzi* proteasome. Chymotrypsin-, trypsin- and caspase-like activities were measured in end point assays for different *T. cruzi* proteasome concentrations (Fig. 6). The β4^R^ mutant proteasome displayed all three enzymatic activities, with apparently lower activity compared to the WT proteasome. For both WT and β4^R^ proteasomes, the caspase activity was slightly higher than the trypsin and chymotrypsin activities. For the Δβ1Δβ2 mutant proteasome only chymotrypsin activity was observed, with, as expected, no detectable turn-over of the β1 and β2 substrates.

**Figure 7:**
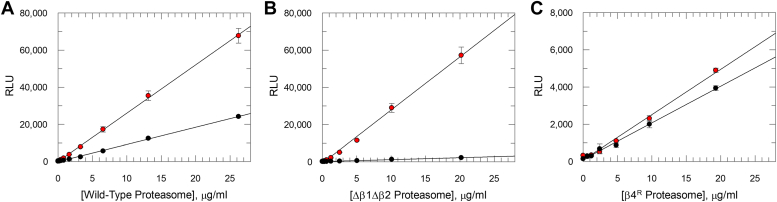
**Chymotrypsin-like activities of recombinant *Trypanosoma cruzi* proteasomes and mutants in the presence and absence of “ES09 Series” inhibitor.** Chymotrypsin activity measured for (*A*) recombinant WT, (*B*) recombinant Δβ1Δβ2 mutant, and (*C*) recombinant β4^R^ mutant *T. cruzi* proteasomes. In all graphs enzyme activities are plotted against *T. cruzi* proteasome concentration either in the presence (*black circles*) or absence (*red circles*) of 1 μM DDD01012248. Data are presented as mean RLU ± SD (*n* = 3 technical replicates). *Solid lines* are linear regression of the data. In all cases correlation coefficient > 0.99. RLU, relative luminescence unit.

Further characterization of the mutant *T. cruzi* proteasomes was achieved by measuring chymotrypsin-, trypsin- and caspase-like activities in the presence and absence of DDD01012248, a kinetoplastid selective “ES09 Series” proteasome inhibitor (Table S3). This compound is highly selective for the β5 active site. DDD01012248 showed partial inhibition of the β5 chymotrypsin enzyme activity of the WT *T. cruzi* proteasome (Fig. 7*A*). Previously we observed the same behavior for oprozomib and hypothesized that the suc-Leu-Leu-Val-Tyr-aminoluciferin substrate was not specific for the chymotrypsin-like active site of the *T. cruzi* proteasome and that residual turnover of the substrate by the trypsin- and/or caspase-like active sites may result in incomplete apparent inhibition of chymotrypsin-like activity (24). To test this, we assessed β5 chymotrypsin inhibition in the Δβ1Δβ2 mutant. Here, full inhibition of the β5 chymotrypsin activity was observed, in line with the residual proteolytic activity of the WT proteasome resulting from β5 substrate turnover by the β1 and/or β2 active sites (Fig. 7*B*). We next tested DDD01012248 against the chymotrypsin activity of the β4^R^ mutant proteasome, and as expected no notable inhibition was detected (Fig. 7*C*). The compound also did not inhibit the trypsin and caspase activities of the β4^R^ mutant, confirming the high level of selectivity in this series (Fig. S4, Table S4).

### Pharmacological validation of mutant *T. cruzi* proteasomes

**Figure 8:**
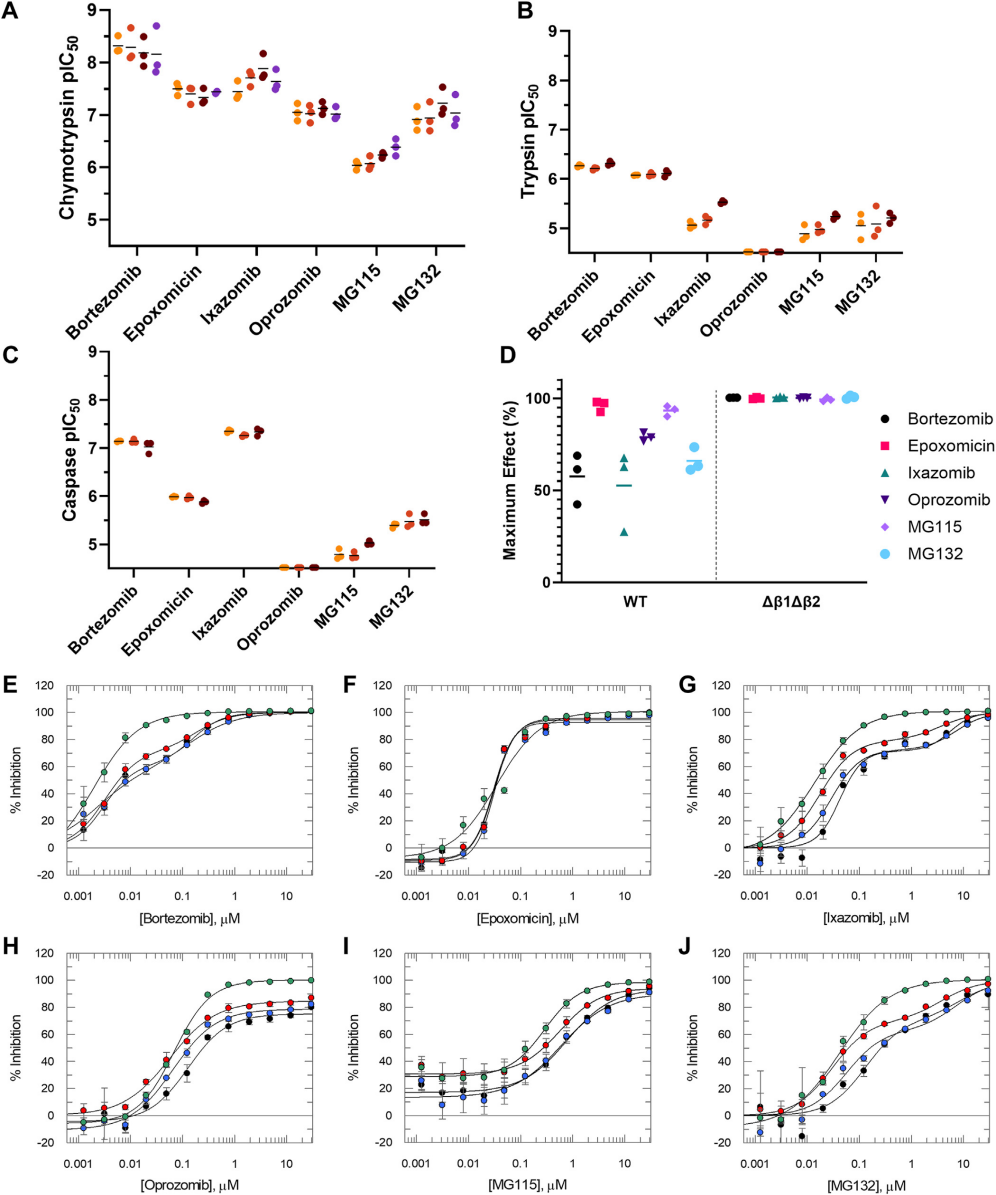
**Inhibition of chymotrypsin-, trypsin-, and caspase-like activities of native and recombinant proteasomes.** Inhibition of (*A*) the chymotrypsin assay, (*B*) the trypsin assay, and (*C*) the caspase assay for “Proteasome Reference Panel” compounds for native (*light orange*), recombinant WT (*dark orange*), recombinant β4^R^ mutant (*brown*), and recombinant Δβ1Δβ2 mutant (*purple*) *Trypanosoma cruzi* proteasomes. Individual pIC_50_ data points (from *N* = 3 independent biological replicates) are shown. Mean chymotrypsin, trypsin, and caspase pIC_50_ data for these compounds, from the independent biological replicates (*N* = 3), can be found in Table S4. Data points with pIC_50_ < 4.52 are plotted on the *x*-axis. *D*, maximum % inhibition plateaus in the chymotrypsin assay for “Proteasome Reference Panel” compounds for recombinant WT and recombinant Δβ1Δβ2 mutant *T. cruzi* proteasomes. Individual maximum % inhibition plateau data (from *N* = 3 independent biological replicates) are shown. For WT biphasic curves (bortezomib, ixazomib, and MG132), the midplateau value is plotted, which represents the maximum level of inhibition of the chymotrypsin activity. Mean chymotrypsin maximum % inhibition plateau data for these compounds, from the independent biological replicates (*N* = 3), can be found in Table S5. *E*–*J*, representative chymotrypsin assay dose-response curves for “Proteasome Reference Panel” compounds against native (*black*), recombinant WT (*blue*), recombinant β4^R^ mutant (*red*), and recombinant Δβ1Δβ2 mutant (*green*) *T. cruzi* proteasomes. In all graphs data are presented as mean % inhibition ± SD (*n* = 3 technical replicates). Data were fitted to a 4-parameter logistic fit model (Equation 2 in Experimental procedures) or, for bortezomib, ixazomib, and MG132 (*black*, *blue*, and *red curves*) a biphasic fit model (Equation 3 in Experimental procedures).

To fully pharmacologically validate the mutant *T. cruzi* proteasomes, the “Proteasome Reference Panel” was tested in the chymotrypsin, trypsin, and caspase assays for four different proteasome samples—native, recombinant WT, β4^R^ mutant, and Δβ1Δβ2 mutant. pIC_50_ data for this reference panel from all three activity assays against all four proteasome samples are shown in Figure 8 and Table S4 (N.B. there is no trypsin or caspase data for the Δβ1Δβ2 mutant, as this mutant has no trypsin or caspase activity). Figure 8, *E*–*J* displays representative dose-response curves for all reference compounds against all proteasome samples in the chymotrypsin assay. Representative dose-response curves for the trypsin and caspase assays are shown in Figs. S5 and S6.

For the six compounds from our “Proteasome Reference Panel” (bortezomib, epoxomicin, ixazomib, oprozomib, MG115, and MG132), highly comparable chymotrypsin-site dose-response curves are observed for the native, recombinant WT and β4^R^ mutant proteasomes. In some cases (bortezomib, ixazomib, and MG132), compounds displayed biphasic behavior, therefore data were fitted to a biphasic fit model (Equations 3 or 4 in Experimental procedures) rather than the typical 4-parameter logistic fit model (Equation 2 in Experimental procedures). The need for a biphasic fit reflects the ability of these compounds to also inhibit the trypsin and caspase sites of the proteasome. and since the chymotrypsin substrate (Suc-Leu-Leu-Val-Tyr-aminoluciferin) is also a substrate for the trypsin and caspase active sites (see above), more than one IC_50_ is being measured in the chymotrypsin assay. In the Δβ1Δβ2 mutant the chymotrypsin substrate can only be turned over by the β5 site, and as expected this leads to clear monophasic dose-response curves for all compounds. When oprozomib, a highly selective chymotrypsin activity inhibitor (46), is tested against the native, recombinant WT and β4^R^ mutant, a partial inhibition profile is observed with the top of the curves < 100% inhibition. As previously described, the remaining residual activity seen is attributed to the chymotrypsin substrate (Suc-Leu-Leu-Val-Tyr-aminoluciferin) also being a substrate for the trypsin and caspase active sites, which are not inhibited by oprozomib. Importantly, oprozomib achieves full inhibition when tested against the Δβ1Δβ2 mutant (which has no trypsin-like or caspase-like activities), confirming full inhibition of the chymotrypsin site, and the ability of the trypsin and caspase active sites to turn over the chymotrypsin substrate (Fig. 8, *D* and *H*). Likewise, all other compounds in the panel also achieved full inhibition of the chymotrypsin activity against the Δβ1Δβ2 mutant. Maximum inhibition for bortezomib, ixazomib, and MG132 (biphasic against the WT proteasome), is observed in the Δβ1Δβ2 mutant at concentrations corresponding to the midplateau of the WT biphasic curves, confirming that the first phase of the biphasic curves represents inhibition of the chymotrypsin active site (Fig. 8, *E*, *G* and *J*).

**Figure 9:**
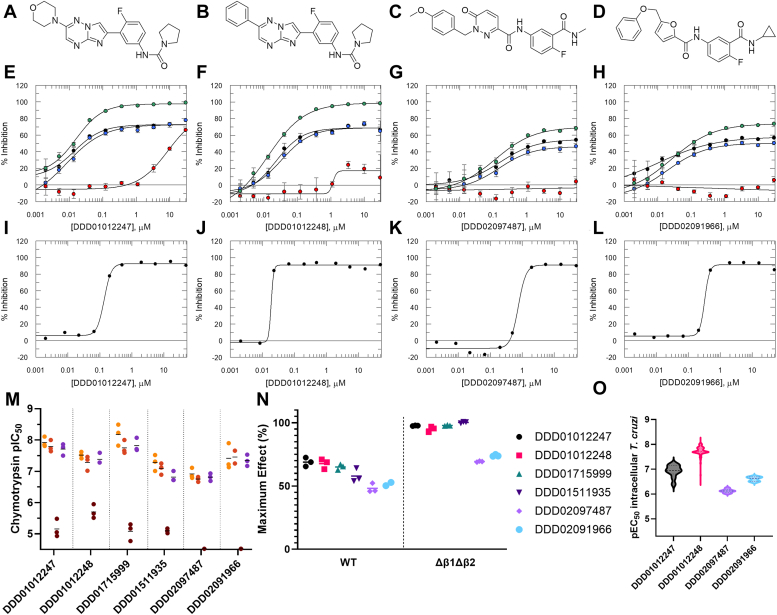
**Profiles of in-house inhibitors against *Trypanosoma cruzi* proteasomes and *T. cruzi* parasites.***A*–*D*, structures of “ES09 Series” compounds DDD01012247 (*A*) and DDD01012248 (*B*), and compounds DDD02097487 (*C*) and DDD02091966 (*D*). See Table S3 and Supplementary Methods. *E*–*H*, representative chymotrypsin assay dose-response curves for DDD01012247, DDD01012248, DDD02097487, and DDD02091966 against native (*black*), recombinant WT (*blue*), recombinant β4^R^ mutant (*red*), and recombinant Δβ1Δβ2 mutant (*green*) *T. cruzi* proteasomes. Data are presented as mean % inhibition ± SD (*n* = 3 technical replicates). Data for independent replicates shown in panel M with mean pIC_50_ data reported in Table S4. *I*–*L*, representative dose-response curves (*n* = 1) for DDD01012247, DDD01012248, DDD02097487 and DDD02091966 against intracellular *T. cruzi* parasites. Data for all replicates shown in panel O. For panels *E*–*L*, all data were fitted to the 4-parameter logistic fit model (Equation 2 in Experimental procedures). *M*, replicate potency data against native (*light orange*), recombinant WT (*dark orange*), recombinant β4^R^ mutant (*brown*), and recombinant Δβ1Δβ2 mutant (*purple*). (*N* = 3 biological replicates, except *N* = 2 for DDD02091966 against recombinant WT). Data points with pIC_50_ < 4.52 are plotted on the *x*-axis. Representative curves for DDD01715999 and DDD01511935 are shown in Fig. S8. *N*, maximum % inhibition plateaus for recombinant WT and recombinant Δβ1Δβ2 mutant proteasome assays. (*N* = 3 biological replicates, except *N* = 2 for DDD02091966 against recombinant WT). For the DDD01511935 WT biphasic curve, the midplateau value is plotted, which represents the maximum level of inhibition of the chymotrypsin activity. *O*, violin plot of potency data against intracellular *T. cruzi* parasites *N* ≥ 5). Mean pEC_50_, standard deviation and *N* values are: DDD01012247 [6.9, 0.2, 10], DDD01012248 [7.7, 0.2, 1095], DDD02097487 [6.1, 0.1, 10] and DDD02091966 [6.6, 0.1, 5].

We further tested the compounds from our own programmes (Fig. 9, *A*–*D*, Table S3). All showed highly comparable dose-response curves for the native and recombinant WT proteasomes, with <100% inhibition at the top plateau (Fig. 9, *E*–*H*, *M* and *N*). For the “ES09 Series” compounds, the remaining residual activity appears due to the chymotrypsin substrate also being turned over by the trypsin and caspase active sites, as described for oprozomib. When the trypsin and caspase sites are mutated in the Δβ1Δβ2 mutant, full inhibition is observed for these compounds (Fig. 9, *E*, *F* and *N* and Table S5). In contrast, the top plateaus remained substantially below 100% for DDD02097487 and DDD02091966 in the Δβ1Δβ2 mutant, indicating that, unlike the “ES09 Series” compounds, these compounds are unable to fully inhibit the *T. cruzi* proteasome chymotrypsin activity (Fig. 9, *G*, *H* and *N* and Table S5). As expected, the β4^R^ mutant proteasome showed resistance to the “ES09 Series” compounds, with an approximate 2-log unit increase in IC_50_. Resistance was also seen for both DDD02097487 and DDD02091966, with >2-log unit increases in IC_50_. Like the “ES09 Series” (12, 13) and DDD02097487 (42), DDD02091966 is also highly selective for the parasite proteasome, with no inhibition of the human chymotrypsin activity observed (Fig. S7).

Finally, we tested the activity of our three in-house series against intracellular *T. cruzi* parasites, and all were active in this assay (Fig. 9, *I*–*L*, *O*).

## Discussion

To enable structure-based drug discovery for the *T. cruzi* proteasome we have expressed and purified recombinant 20S *T. cruzi* proteasomes using the biGBac system. We observed catalytic activity for all three active sites and pharmacological profiling with a range of inhibitors, covering multiple modes of inhibition, returned very comparable results between the native and recombinant *T. cruzi* proteasomes supporting the use of the recombinant proteasomes for Chagas disease drug discovery efforts. While pharmacologically they were comparable, we did find a difference in relative activities of the three catalytic sites. For the native proteasome all three activities were equivalent, whereas the recombinant proteasomes exhibited different rates of turnover for the different substrates, with caspase substrate turnover the fastest. Cleavage of the three active site propeptides occurs after assembly of the full core particle, through an autocatalytic activation mechanism (47). Potentially, incomplete and differential activation of the three sites in the recombinant proteasome leads to the differential cleavage rates (catalytic subunit propeptides were detected in the recombinant proteasome sample, Table S1, but not seen in the CryoEM electron density). The absence of *T. cruzi* chaperones supporting autoactivation (*e.g.* Ump1), may underlie less effective autocatalysis.

In addition to the aforementioned advantages of recombinant expression, the ability to rapidly generate specific mutant proteasomes provides fast access to key tools for structure-function understanding and drug discovery. This is particularly valuable for deconvoluting selective inhibition of the three active sites, for working with proteasome mutations that affect cell viability, for high-throughput drug discovery screening and when working with organisms where single nucleotide gene-editing is challenging, such as *T. cruzi*. We generated two mutant proteasomes. The mutations in the β4^R^ recombinant *T. cruzi* proteasomes were selected based on previous resistance generation experiments with the “ES09 Series.” While activity of the β4^R^ proteasome was lower than that of the WT proteasome, indicating that the mutations affect proteasome assembly or function, sufficient activity remained to assess compound potencies. As expected, resistance was seen for all exemplars of this series and for the pyridazinone DDD02097487 (42). Resistance was also observed for our new *T. cruzi* proteasome inhibitor, DDD02091966, indicating that this compound binds in a similar way. In contrast, WT sensitivity to classical peptidic proteasome inhibitors, which have a different binding mode, was retained. When assessing new proteasome inhibitors, it is important to understand if their binding mode is similar or different to known inhibitors and the β4^R^ proteasome thus provides a rapid tool to assess cross-resistance and binding mode similarity for new inhibitors.

Proteasomes without β1/β2 catalytic activity proved very valuable to understand catalytic site selectivity. Our work demonstrates that the standard commercial chymotrypsin-site (β5) substrate (suc-Leu-Leu-Val-Tyr-aminoluciferin) is also cleaved by the β1 and/or β2 active sites. This is in line with pharmacological evidence for the *P. falciparum* proteasome, where application of a β2-specific inhibitor suggested that suc-Leu-Leu-Val-Tyr-AMC was also turned over by the β2 active site (48). Substrate selection is governed by the interactions between the substrate and the active site S1 selectivity pocket. The ability of the β1 and/or β2 active sites to cleave suc-Leu-Leu-Val-Tyr-aminoluciferin thus indicates that this sequence is compatible with the β1 and/or β2 active site(s) S1 selectivity pockets. The binding of the peptidic inhibitor MG132 (carbobenzoxy-l-leucyl-l-leucyl-l-leucinal) to all three human active sites (and the inhibition of all 3 *T. cruzi* active sites by MG132, Table S4), provides precedent for the recognition of short hydrophobic sequences by the β1 and β2 active sites (49). Where site-specific proteolysis is being investigated, it is thus critical to either use the relevant active site mutant proteasomes or more selective substrates (20, 50).

Substrate selectivity is of significant importance for the interpretation of the maximum level of inhibition seen with inhibitors, in particular those that are highly selective for the β5 site. Using the standard substrates, such inhibitors show partial inhibition, not because they do not fully inhibit the β5 activity, but because the assay substrate is turned over by the uninhibited β1 and/or β2 sites. Mutants with inactivated β1 and β2 sites retain β5 activity, and can thus be used to accurately interrogate the ability of inhibitors to fully inhibit the β5 site. We assessed our in-house series of proteasome inhibitors, including the “ES09 Series”, the pyridazinone DDD02097487, and a novel chymotrypsin-selective *T. cruzi* proteasome inhibitor (DDD02091966) (Table S3) against the Δβ1Δβ2 proteasome. While all these compounds bind to the same area of the proteasome (based on previously reported structures and their resistance profile against the β4^R^ proteasome), the “ES09 Series” compounds achieved full inhibition of the Δβ1Δβ2 mutant, whereas DDD02097487 and DDD02091966 could only partially inhibit the chymotrypsin activity in the Δβ1Δβ2 mutant. This mutant thus allows precise identification of site-selective partial inhibitors. Why the latter two compounds cannot fully inhibit the chymotrypsin activity remains to be further investigated. The benzyl moiety of DDD02097487 occupies a hydrophobic pocket at the β4/β5 interface that is not engaged by the “ES09 Series” compounds (42), and potentially this difference in binding mode affects the mechanism of inhibition. Interestingly, in spite of only partially inhibiting the *T. cruzi* proteasome, these compounds remain potent inhibitors of intracellular *T. cruzi* growth, indicating that the proteasome is a highly vulnerable target (51), with full inhibition not required for an antiparasitic effect. Whether partial proteasome inhibition may have more subtle effects, for example on the ability to clear less susceptible or dormant subpopulations remains to be determined (52, 53).

The mutant proteasomes presented here thus provide a toolbox for the pharmacological study of the *T. cruzi* proteasome. In drug discovery, the primary screening tool will depend on the purpose of the campaign. The Δβ1Δβ2 mutant can be used to identify full inhibitors of the chymotrypsin activity, the β4^R^ proteasome can be used to identify new chemical matter that inhibits through a different mechanism than all reported selective *T. cruzi* proteasome inhibitors, and the WT proteasome can be used to identify compounds that target all three active sites.

Structural information is critical to guide drug discovery. Here, we report the structure of the *T. cruzi* proteasome. We generated CryoEM structures for the apo forms of both the native *T. cruzi* proteasome, purified from the parasite, and the recombinant proteasome. The structures are highly similar, further confirming the suitability of the recombinant proteasome for drug discovery and other studies. Comparison with previously published apo and liganded structures of the *L*. *tarentolae* proteasome shows a high level of similarity between the *L. tarentolae* and *T. cruzi* structures, and also reveals that two residues in the apo *T. cruzi* structure (His 233 and Phe 236) adopt a conformation similar to the conformation in the liganded *L. tarentolae* structure (but not the apo *L. tarentolae* structure). This observation suggests that the *T. cruzi* apo proteasome structure could provide a more relevant starting model for ligand virtual screening than the *L. tarentolae* apo proteasome. In addition, the reliable placement of >1000 water molecules in the recombinant structure is highly relevant for drug discovery, as water molecules often play a crucial role in protein-ligand binding (54). The high-resolution structure will therefore enable improved computational predictions for protein-ligand interactions. Comparison with other protozoan proteasome structures and the human structure suggests that the hydrophobic β4/β5 selectivity pocket is a unique feature of kinetoplastid organisms. In *P. falciparum*, another medically important protozoan parasite, two polar amino acids occupy this pocket. Accordingly, all structurally confirmed *P. falciparum* proteasome inhibitors bind to different pockets in the β5 subunit (50, 55, 56, 57). Furthermore, the comparison revealed a potential channel or groove connecting the *T. cruzi* β1 and β2 active sites that appears not to be present in the human proteasome. In the future, new mutant recombinant proteasomes could be generated to investigate the relevance of this groove with respect to proteasome function.

Finally, as mentioned above, we present a new 5-(phenoxymethyl)furan-2-carboxamide-based proteasome inhibitor of the *T. cruzi* proteasome (DDD02091966). This compound is the result of a medicinal chemistry programme where we applied our understanding of *T. cruzi* proteasome inhibitor structure-activity relationships (gained from the ES09 and pyridazinone series) to a high-throughput screening hit with a furan core. DDD02091966 is a highly selective inhibitor of the *T. cruzi* proteasome chymotrypsin activity with promising potency against intracellular *T. cruzi* parasites. As such, this compound is an interesting new tool for studying the *T. cruzi* proteasome and a potential new starting point for drug discovery for Chagas disease.

The work presented here provides structural insights into the *T. cruzi* proteasome, explains how partial substrate selectivity impacts pharmacological outputs and enables proteasome drug discovery for Chagas disease, by overcoming the challenges associated with purifying native proteasomes from the parasites and by reporting a new highly selective inhibitor. Very few validated drug targets are available for Chagas disease drug discovery, so efforts to make these targets more accessible are critical. Our approaches are applicable to other neglected tropical diseases and could provide major benefits where similarly few validated targets exist, such as parasitic worms.

## Experimental procedures

### Recombinant proteasome cloning

The 14 subunits (α1-7 and β1-7) of the *T. cruzi* proteasome were PCR amplified from genomic DNA (*T. cruzi* strain Silvio X10/7 (58)) or purchased as gene blocks (IDT DNA) and subcloned into PCR blunt vectors (Invitrogen) prior to sequencing to confirm their identify. Subunit α1 was recloned to have a C-terminal His10-tag based on previous CryoEM data of the *L. tarentolae* showing it pushing away from the body of the structure (13).

Cloning of the *T. cruzi* proteasome was carried out using the MultiBac-derived biGBac system for Gibson cloning (34). Subunits were PCR amplified by touchdown PCR with Herculase II Fusion polymerase (Agilent) using suitable Cas1-Cas5 primers (IDT DNA) and analyzed on 1% agarose gels. Correct sized bands were gel-extracted using minElute columns (QIAGEN). Briefly, 10 μg of pBIG1 vector was digested with *Swa*I (NEB) to linearize and assembled with inserts using HiFi DNA assembly (New England Biolabs (NEB)) and transformed into chemically competent DH5 alpha cells (NEB) following manufacturers protocols and plated onto LB agar plates supplemented with ampicillin. Subunits α1-5 were cloned into pBIG1A, α6 and α7 and β1-3 into pBIG1B, β4 and β5 into pBIG1C, and β6 and β7 into pBIG1D. Colonies were miniprepped to extract DNA using QIAGEN miniprep kits, digested using *Pme*I (NEB) and run on a 1% agarose gel to confirm the correct sized inserts. Those plasmids displaying the correct number of appropriately sized bands were subsequently sequenced by the DNA sequencing services Dundee.

### Mutagenesis

Point mutations were created in individual subunits using overlapping PCR from the original templates using Polh and Tn7L primers outside the genes and suitable internal primers to introduce selected mutations. For Δβ1Δβ2, β1 Thr 55, and β2 Thr 67 were both mutated to Ala, the β4^R^ mutant was produced by mutating Phe 24 and Ile 29 to Leu and Met respectively in β4 subunit.

Mutated subunits were assembled to create pBIG1B (α6-7, Δβ1, Δβ2, and β3) and pBIG1C (β4^R^, β5) using HiFi builder (NEB) and sequenced to confirm mutations within the mutant subunits and to check the other subunits in the respective pBIG vectors.

### Recombinant proteasome expression and purification

Once confirmed, all pBIG plasmids were transformed into EmBacY cells (Geneva Bioscience) by heat shock and positive clones selected by blue/white selection. Positive clones were grown in 10 ml insect cell media and bacmid DNA extracted using isopropanol precipitation prior to transfection into Sf9 cells. Confluent Sf9 cells were diluted using SF900III media (Gibco), and 2 × 10^5^ cells allowed to adhere to wells in a 24-well tissue culture plate for 45 min. A total of 5 μl of bacmid DNA was added to 100 μl SF900III media and separately 2 μl Insect GeneJuice Transfection Reagent (Novagen) was added to 100 μl SF900III media, vortexed, and allowed to rest for 5 min prior to mixing together and leaving for 45 min. The media were aspirated from the adhered cells and replaced with the bacmid-SF900III-transfection reagent mixture for 24 h, prior to being topped up with 800 μl SF900III media supplemented with 100 U/ml Penicillin-Streptomycin (Thermo Fisher Scientific) and 2 mM glutamine (Gln). The cultures were incubated for further 5 days in the dark at 27 °C to produce P0 virus.

Initial P0-P1 cultures were carried out in 3 ml in 24-well deep well blocks (Greiner) covered with Breathe-Easy seals (Greiner) seeded with 2 × 10^6^ cells per ml in SF900III media (supplemented with Pen-Strep and Gln) using 120 μl P0 virus to confirm transfection had been successful. Subsequently, differing ratios of each pBIG1A-D virus were mixed and repeat P0-P1 test expressions carried out with the best ratio being an excess of pBIG1A (A:B:C:D - 2:1:1:1).

Large scale growths were carried out by amplifying P0 virus through to P2 in 200 ml. For expression, confluent Sf9 cells incubated in SF900III media (supplemented with Pen-Strep and Gln) were diluted to 1.5 × 10^6^ cells/ml in 1 l, inoculated with 2.5% virus P2 and grown for 48 h in 500 ml in 2 l flasks at 26.5 °C, 135 rpm. Cells were pelleted, resuspended in buffer A (25 mM Hepes, 500 mM NaCl, 20 mM imidazole, 5% glycerol, 0.5 mM tris(2-carboxyethyl)phosphine (TCEP), pH 7.5, supplemented with DNAse and protease inhibitors (Roche Complete) and lysed using a constant cell disruptor (30KPSI). Cell debris was pelleted by centrifugation (40,000 g for 30 min at 4 °C) and supernatant filtered (0.2) μm prior to loading onto a HisTrap HP 5 ml column (Cytiva) preequilibrated in buffer A using an AKTA chromatography system (Cytiva). Protein was eluted over 20 column volumes with a gradient of 5 to 100% buffer B (25 mM Hepes, 500 mM NaCl, 500 mM imidazole, 5% glycerol, 0.5 mM TCEP pH 7.5). Fractions containing the proteasome were concentrated to ∼1 ml and buffer exchanged to buffer C (25 mM Hepes, 150 mM NaCl, 5% glycerol, 0.5 mM TCEP pH 7.5) and flash frozen for assay. Identity of the *T. cruzi* proteasome subunits was confirmed by in-solution digestion with trypsin and analysis by LC-MS/MS by the FingerPrints Proteomics Service (University of Dundee).

For SDS-PAGE analysis all samples were run on Bio-Rad TGX gels with Precision Plus Protein markers (Bio-Rad) and imaged on Bio-Rad ChemiDoc following manufactures protocols. Western blots were performed by blocking gel in 5% milk PBS tween 20, for 1 h, followed by blotting with His Tag Horseradish Peroxidase-conjugated Antibody (R&D systems Catalog#: MAB050H. The specificity of the antibody was tested by the manufacturer, see website for examples) for 1 h, prior to washing 3x PBS tween and incubating with Clarity Max Western ECL Substrate (Bio-Rad). Silver staining was carried out using Pierce Silver Stain for Mass Spectrometry (Pierce) following manufacturers protocols.

Concentration of each of the recombinant proteasome samples was measured using extinction coefficients for all subunits of the *T.cruzi* proteasome on a Denovix DS11+ spectrometer after blanking against buffer C (25 mM Hepes, 150 mM NaCl, 5% glycerol, 0.1 mM TCEP pH 7.5), in triplicate. Samples were determined to be over 95% pure based on densitometry using silver stain as determined by Bio-Rad Image Lab (https://www.bio-rad.com/en-uk/product/image-lab-software), based on 2 μg loaded protein.

### Mass spectrometry protein identification

Purified recombinant *T. cruzi* proteasome samples were dried in a speed vac prior to being resuspended and incubated with 8 M urea, 0.02 M DTT in 0.2 M ammonium bicarbonate for 2 h at 30 °C. Subsequently, 5 μl of 0.1 M iodoacetamide was added, and the sample incubated on a shaker in the dark for a further 30 min prior to 1:10 dilution of sample in MilliQ water. Briefly, 1 μl of 1 mg/ml trypsin was added, and samples were incubated for 16 h at 30 °C prior to acidification and analysis using an UltiMate 3000 RSLCnano system (Thermo Fisher Scientific) coupled to a Q Exactive Plus Mass Spectrometer (Thermo Fisher Scientific).

Subsequently, 15 μl samples were injected and washed on the C18 trap column with 0.1% formic acid (Loading Buffer). After 5 min a gradient was formed with buffers A and B (Buffer A: 0.1% formic acid, Buffer B: 80% acetonitrile in 0.1% formic acid) over a 20 min gradient at 0.3 μl/min.

Peptides were initially trapped on an Acclaim PepMap 100 (C18, 100 μM × 2 cm) and then separated on an Easy-Spray PepMap RSLC C18 column (75 μM × 50 cm) (Thermo Fisher Scientific). Samples were transferred to the mass spectrometer *via* an Easy-Spray source with temperature set at 50 °C and a source voltage of 2.0 kV.

Q Exactive Plus: Top 15 Method: 1 MS plus 15 MS/MS (150 min acquisition). Operating in data-dependent acquisition mode with a lock mass of 455.120024. Full MS1 scans were performed at 70,000 resolution, followed by 15 sequential dependent MS2 scans where the top 15 ions were selected for collision-induced dissociation (normalized collision energy NCE = 35.0) and analysis in the Ion Trap with an MSn AGC target of 5000. An isolation window of 2.0 m/z units around the precursor was used, and selected ions were then dynamically excluded from further analysis.

Data were analyzed by Q Exactive Plus. RAW files were converted to MSF files (Proteome Discoverer Version 2.2; https://www.thermofisher.com/uk/en/home/industrial/mass-spectrometry/liquid-chromatography-mass-spectrometry-lc-ms/lc-ms-software/multi-omics-data-analysis/proteome-discoverer-software.html). Extracted data then searched against *T. cruzi* proteasome subunit peptides and database version Mascot Search Engine (Version 2.6.2; https://www.matrixscience.com/server.html). Mass spectrometry analysis was carried out at the “FingerPrints” Proteomics Facility, School of Life Sciences, University of Dundee.

Native proteasome purification was carried out as previously described in Zmuda *et al.* (24).

### CryoEM structure determination

#### CryoEM sample preparation and data collection

The native *T. cruzi* apo proteasome in 50 mM Tris pH 7.5, 150 mM NaCl, 100 mM sucrose, 5 mM MgCl_2_, 2 mM ATP, 1 mM DTT, and 1 mM EDTA was concentrated using a 100K Vivaspin concentrator to a volume of ∼5 to 8 μl (concentration not measured). The recombinant *T. cruzi* apo proteasome in 20 mM Hepes pH 7.5, 150 mM NaCl, 0.5 mM TCEP, and 5% glycerol, (2.35 mg/ml as measured by Nanodrop A280) was diluted 1:1 with a dilution buffer of 20 mM Hepes pH 7.3, 150 mM NaCl. For both samples, Quantifoil R 1.2/1.3 (Cu 300) with 3 nm carbon grids were freshly glow discharged using a Pelco easiGlow at 25 mA for 30 s. A Vitrobot Mark IV (Thermo Fisher Scientific) was used at 4 °C and 100% humidity for double-sided blotting (2.5 s, single application) and plunging grids into liquid ethane.

High resolution datasets were collected on a Titan Krios electron microscope equipped with a Gatan K3 direct electron detector at the Cambridge Pharmaceutical CryoEM Consortium, Nanoscience Centre, University of Cambridge. Data collection parameters are given in Table S6. Data processing was performed using Relion 3.1 (59), initially carrying out motion correction, contrast transfer function (CTF) estimation and autopicking (Laplacian of Gaussian). The subsequent steps are described separately for each dataset.

#### CryoEM native dataset processing

The dataset consisted of 7200 movies, exhibiting a good mix of side view, top view, and intermediate view proteasome particles. After particle extraction and several rounds of 2D classification, 214,900 particles were selected. Several particle classes were observed that were clearly not from proteasome. A previously determined high resolution *L. tarentolae* proteasome CryoEM structure (unpublished) was used as the reference for 3D classification (4 classes) using C1 symmetry. After selecting the best particle classes, 3D autorefinement was run with the resulting 171,041 particles, leading to a map at 3.2 Å resolution. The map was aligned for C2 symmetry, and a further 3D classification was carried out with C2 symmetry (3 classes). A single class of 50,663 particles was selected for 3D autorefinement giving an indicated resolution of 3.23 Å. After generation of a mask and post processing, the resolution had increased to 2.64 Å. A lengthy CTF refinement and particle polishing protocol was then run which yielded a final map resolution of 2.31 Å (Fig. S9).

#### CryoEM recombinant dataset processing

The dataset consisted of 11,666 movies and was dominated by side view proteasome particles. After particle extraction and several rounds of 2D classification, 113,240 particles were selected and used to generate an initial model (using C1 symmetry), which was then used as a reference for 3D classification (4 classes). The best resulting 3D class (58,543 particles) was then aligned for C2 symmetry and used as the reference for 3D classification (4 classes) with C2 symmetry using the 113,240 particle set. The best resulting 3D class (92,013 particles) was then run through 3D auto-refinement giving an indicated resolution of 3.52 Å. After generation of a mask and post-processing, the resolution had increased to 3.17 Å. A lengthy CTF refinement and particle polishing protocol was then run which yielded a final map resolution of 2.25 Å (Fig. S10).

#### CryoEM model building

Model building was carried out using the CCP4 program suite (60). Initial coordinates for the proteasome model were obtained using Molecular Replacement with Molrep (61) using an unpublished high resolution *L. tarentolae* CryoEM proteasome structure as a search model. It was apparent that the quality of the recombinant proteasome map was considerably higher than that of the native proteasome map. In the case of the recombinant proteasome final CTF refined/particle polished map at 2.25 Å, locations where the protein sequences differed between *L. tarentolae* and *T. cruzi* proteasome were, in general, readily apparent and so this structure was built first using Coot (https://www2.mrc-lmb.cam.ac.uk/personal/pemsley/coot/) (62).

One half of the proteasome barrel (chains A-N) was adjusted by rigid body fitting of the individual protein chains to the map. Amino acid substitutions were made in Coot, guided by sequence alignments of the *L. tarentolae* and *T. cruzi* proteasome sequences. The other half of the model (chains O-b) was generated using the C2 symmetry of the proteasome. Using a model consisting only of the subunits A-N, waters were added using Coot "Find waters" with the default settings (1.8 RMSD, 2.4 Å min, and 3.2 Å max) and designated as chain "c". The first half of the proteasome was then transformed on to the O-b subunits and the "new" waters were designated as chain "d". At the regions near the interface between the two halves there were many overlapping waters (duplicates). The duplicate waters were identified in Coot by being very close to "other waters". These were then manually removed in Coot, in all cases the "d" chain water was removed rather than the "c" chain water. The structure was refined using Refmac (63) using restrained refinement and two-fold noncrystallographic symmetry restraints.

The refined recombinant proteasome structure was used as the starting point for the native proteasome structure. The main map used for model building was the final 2.31 Å CTF refined/particle polished map. However, for checking and adjusting parts of the model, particularly near some areas of solvent, it was helpful to also refer to the final (unsharpened) Refine3D map (produced before the final mask creation and postprocessing stages) and the 2.64 Å map (obtained prior to CTF refinement/particle polishing). Some differences were expected in a few amino acids compared to the recombinant protein sequences: for example, the C terminus of the recombinant α1 subunit ends with a His tag. However, there was only one location where there was an expected amino acid difference in a region where the map quality allowed model atoms to be built: the α7 subunit in the recombinant protein was Tyr 139, while the native protein was expected to be Cys 139. The electron density for this residue was insufficiently distinct to suggest either possibility convincingly so the residue was left unchanged (as Tyr 139). As the electron density was generally not as clear in many regions as seen in the recombinant protein there were not many substantial changes made in this model. While some of the density, particularly near the solvent regions, was relatively poor, much of the density was very good and so water molecules were added following the same protocol as for the recombinant structure determination.

Refinement details for the two final models are given in Table S7. The electron microscopy maps and structure coordinates have been deposited in the Protein Data Bank (PDB ID: 9F9T, EMDB ID: EMD-50260 and PDB ID: 9F9P, EMDB ID: EMD-50258).

### Bioinformatics

Variation of RMSD across proteasome subunits and species was determined using UCSF ChimeraX-1.7.1 (https://www.cgl.ucsf.edu/chimerax/) (64). Percentage sequence identity was calculated using Clustal Omega on the EBI server and visualized as multiple sequence alignments using JalView (https://www.jalview.org/) (65, 66). Structural figures produced using The PyMOL Molecular Graphics System, Version 3.0.3 Schrödinger, LLC (https://www.pymol.org/).

### *T. cruzi* biochemical proteasome assays

Chymotrypsin-, trypsin-, and caspase-like activity assays were performed using a commercially available Proteasome-Glo 3-substrate system assay kit (catalog number G8532) (Promega Corporation) according to the manufacturers protocols and as previously described (24).

Prior to running assays, Proteasome-Glo buffer was used to reconstitute the luciferin detection reagent (containing a recombinant thermostable luciferase enzyme). The resuspended luciferin detection reagent was then mixed with Proteasome-Glo chymotrypsin-, trypsin- or caspase-like substrates (either 40 μM suc-Leu-Leu-Val-Tyr-aminoluciferin, 30 μM Z-Leu-Arg-Arg-aminoluciferin (Z is benzyloxycarbonyl) or 40 μM Z-Nle-Pro-Nle-Asp-aminoluciferin respectively (these concentrations represent 2x final assay concentrations)). These substrate mixtures were allowed to incubate at room temperature for 60 min prior to use to allow the removal of any contaminating free aminoluciferin.

Proteasome assays were carried out using 384-well, white, low-volume plates (catalog number 784904) (Greiner Bio-One) at room temperature (∼23 °C) in 8 μl reaction volumes. When activity checks were to be run in the presence or absence of a fixed concentration of proteasome inhibitor, 80 nL of the inhibitor (at a concentration 100x the required final assay concentration), or 80 nL dimethylsulfoxide (DMSO), were dispensed into assay plates using an Echo 550 acoustic dispenser (Labcyte).

A 1 in 2 dilution series of the various *T. cruzi* proteasome samples (native, recombinant WT, β4^R^ mutant, and Δβ1Δβ2 mutant) were produced in assay buffer (50 mM Tris–HCl, pH 7.5, 10 mM sucrose, 5 mM MgCl_2_, 1 mM DTT, 2 mM ATP, 150 mM NaCl, 1 mM EDTA, and 0.05 mg/ml bovine serum albumin). Subsequently, 4 μl of the various concentrations of diluted proteasome were added to assay plates using an electronic multichannel pipette (Integra Biosciences Ltd). When kinetic read activity checks were performed (Fig. 2), proteasome final assay concentrations used were 115 μg/ml, 115 μg/ml, and 230 μg/ml native proteasome (in the chymotrypsin, trypsin, and caspase assays, respectively), and 4.1 μg/ml, 3.4 μg/ml, or 1.4 μg/ml recombinant WT proteasome (in the chymotrypsin, trypsin, and caspase assays, respectively). When activity checks were to be run in the presence or absence of a proteasome inhibitor plates containing inhibitor or DMSO control were incubated with the relevant proteasome sample for 60 min at room temperature (this step was not included in activity check experiments where compounds were not used).

Following the 60 min incubation of proteasome and compound (if required), assays were initiated with the addition of 4 μl of substrate mix (containing substrate and the luciferin detection reagent) using an electronic multichannel pipette. Final assay concentrations of substrates used were either 20 μM Suc-Leu-Leu-Val-Tyr-aminoluciferin, 15 μM Z-Leu-Arg-Arg-aminoluciferin, or 20 μM Z-Nle-Pro-Nle-Asp-aminoluciferin for chymotrypsin-, trypsin- or caspase-like activity respectively.

Luminescence was read either kinetically or as an endpoint read using an EnVision 2102 Multilabel Reader (PerkinElmer, Inc) with 0.2 s per well reading time. When kinetic assays were run, luminescence was read every 5 min for 90 min. Endpoint assays were run for 60 min before luminescence was read. All data processing and linear fit analyses were performed using Microsoft Excel for Microsoft 365 software (Redmond, Washington; https://www.microsoft.com/).

### Proteasome inhibition assays

When proteasome inhibitors were to be screened in the chymotrypsin, trypsin, and caspase assays, test compounds were dispensed into assay wells using an ECHO 550 acoustic dispenser (Labcyte) (final DMSO concentration 1%). Compounds were tested in either a 10-point concentration series (30 μM top final assay concentration, 1 in 3 dilution series) or a 12-point concentration series (30 μM top final assay concentration, 1 in 2.5 dilution series). Each assay plate also contained control wells into which either DMSO or bortezomib (final assay concentration 30 μM) were dispensed, with these DMSO and bortezomib control wells representing 0% inhibition and 100% inhibition, respectively.

Proteasome assays were then performed as described above using Promega Proteasome-Glo 3-substrate system assay kit. Specifically, the native, recombinant WT, β4^R^ mutant, and Δβ1Δβ2 mutant proteasome samples were diluted in assay buffer (50 mM Tris–HCl, pH 7.5, 10 mM sucrose, 5 mM MgCl_2_, 1 mM DTT, 2 mM ATP, 150 mM NaCl, 1 mM EDTA, and 0.05 mg/ml bovine serum albumin) to a concentration that corresponded to a chymotrypsin assay signal of ∼12,000 relative luminescence unit (RLU) (as determined each assay day, with proteasome concentrations used ranging from: 180–359 μg/ml native proteasome, 3.8–7.5 μg/ml recombinant WT proteasome, 61.8–77.3 μg/ml β4^R^ proteasome, and 4.2–10.1 μg/ml Δβ1Δβ2 proteasome). Diluted proteasome (4 μl) was added to assay plates using an electronic multichannel pipette (Integra Biosciences Ltd) and the proteasome incubated with compound for 60 min at room temperature.

Following the 60 min incubation of proteasome and compounds, assays were initiated with the addition of 4 μl of a substrate mix (containing substrate and the luciferin detection reagent) using an electronic multichannel pipette. Final assay concentrations of substrates used were either 20 μM Suc-Leu-Leu-Val-Tyr-aminoluciferin, 15 μM Z-Leu-Arg-Arg-aminoluciferin, or 20 μM Z-Nle-Pro-Nle-Asp-aminoluciferin for chymotrypsin-, trypsin-, or caspase-like activity, respectively. Following a further 60 min assay incubation, luminescence was read using an EnVision 2102 Multilabel Reader (PerkinElmer, Inc) with 0.2 s per well reading time.

All data processing was performed using Microsoft Excel for Microsoft 365 software. Based on the mean RLU assay signal of the DMSO (RLU_HIGH_) and bortezomib (RLU_LOW_) control wells on each assay plate, a normalized percentage inhibition (% inhibition) value for each test compound well was determined using Equation 1.

(1) $$\%\;inhibition=100-\left(\frac{\left({RLU}_{\text{TEST}\;\text{COMPOUND}\;}-{RLU}_{\text{LOW}\;}\right)}{\left({RLU}_{\text{HIGH}}-{RLU}_{\text{LOW}\;}\right)}\right)*100$$

Nonlinear dose-response curve fitting was carried out using the Microsoft Excel add-in, XLfit (IDBS). Percentage inhibition data were either fitted to a standard 4-parameter logistic fit model (XLfit model 205; Equation 2) or a biphasic model (either XLfit model 300; Equation 3 or a custom biphasic model; Equation 4 (used in cases where the top and/or bottom parameters deviated from 100%/0% inhibition)).

(2) $$y=A+\frac{\left(B-A\right)}{1+{\left(\frac{C}{x}\right)}^{D}}$$

Where A = % inhibition at bottom, B = % inhibition at top, C = IC_50_, D = slope, *x* = inhibitor concentration and *y* = % inhibition.

(3) $$y=\left(\frac{A}{\left(1+10^{\left(C-\log\left(x\right)\right)*B}\right)}\right)+\left(\frac{\left(100-A\right)}{\left(1+10^{\left(D-\log\left(x\right)\right)*B}\right)}\right)$$

With A = % inhibition at midplateau, B = slope, C = log(IC_50_^A^), D = log(IC_50_^B^)). Inhibition at the bottom of the curve is fixed to 0% and at the top to 100%.

(4) $$y=\mathrm{Min}+\left(\left(\frac{\left(\mathrm{Max}-\mathrm{Min}\right)}{100}\right)*\;\left(\left(\frac{A}{\left(1+10^{\left(C-\log\left(x\right)\right)*B}\right)}\right)+\left(\frac{\left(100-A\right)}{\left(1+10^{\left(D-\log\left(x\right)\right)*B}\right)}\right)\right)\right)$$

Where Max = % inhibition at top of curve, Min = % inhibition at bottom of curve, A = % inhibition at mid-plateau, B = slope, C = log(IC_50_^A^) and D = log(IC_50_^B^).

All IC_50_ data generated were converted to pIC_50_ using Equation 5. Where biphasic dose-response curves were observed, pIC_50_ values for the most potent curve (corresponding to inhibition of the chymotrypsin site) are reported.

(5) $$p{IC}_{50}=-\log\;{IC}_{50}\;\left(M\right)$$

Graphs presented in this manuscript were generated using GraFit v7.0.3 (Erithacus Software, East Grinstead, UK; http://www.erithacus.com/grafit/) or Prism 10.3.1 (GraphPad Software; https://www.graphpad.com/).

Human proteasome assays were carried out as described in (42).

### Intracellular *T. cruzi* assay

Potency against intracellular *T. cruzi* Silvio X10/7 A1 amastigotes was determined as described previously (53), with a sole modification that the compound treatment duration was 96 h instead of 72 h. Vero host cells were obtained from The European Collection of Authenticated Cell Cultures (cat. number 84113001) and tested for *mycoplasma* (negative). *T. cruzi* Silvio X10/7 A1 was obtained from Prof. Alan Fairlamb, and discrete typing unit confirmed by genotyping (53).

### Compounds

Structures for the compounds from our in-house series are shown in Table S3.

## Data availability

All data is available in the main manuscript and the supporting information.

## Supporting information

This article contains supporting Information (12, 13, 42, 66).

## Conflict of interest

The authors declare the following competing financial interest(s): P. R. and E. V. E. hold shares in GSK plc. The other authors declare that they have no conflicts of interest with the contents of this article.
