# Supplementary material for: Pharmacological and structural understanding of the Trypanosoma cruzi proteasome provides key insights for developing site-specific inhibitors
Source: J Biol Chem. 2024 Dec 9;301(1):108049. doi: 10.1016/j.jbc.2024.108049 (PMC11748689; doi:10.1016/j.jbc.2024.108049)
Supplement: Supporting information [file mmc1.docx]

**Supplementary Materials**

Pharmacological and structural understanding of the *Trypanosoma cruzi* proteasome provides key insights for developing site-specific inhibitors

Thomas C. Eadsforth^1^*, Leah S. Torrie^1^*, Paul Rowland^2^, Emma V. Edgar^2^, Lorna M. MacLean^1^, Christy Paterson^1^, David A. Robinson^1^, Sharon M. Shepherd^1^, John Thomas^1^, Michael G. Thomas^1^, David W. Gray^1^, Vincent L. G. Postis^1^ and Manu De Rycker^1%^

*: contributed equally

%: corresponding author: m.derycker@dundee.ac.uk

Affiliations:

1: Wellcome Centre for Anti-Infectives Research, University of Dundee, Dundee, UK, DD1 5EH.

2: GSK, Medicines Research Centre, Gunnels Wood Road, Stevenage, UK, SG1 2NY.

Table of Contents

[Figure S1. Expression and purification of recombinant *T. cruzi* proteasomes 2](#_Toc182397726)

[Figure S2. Final *T. cruzi* proteasome CryoEM maps with refined structures 3](#_Toc182397727)

[Figure S3. Multiple sequence alignment of *T. cruzi*, *T. vaginalis*, *H. sapiens,* *L. tarentolae* and *P. falciparum* catalytic β1, β2 and β5 subunits 7](#_Toc182397728)

[Figure S4. Trypsin- and caspase-like activities of recombinant wild-type and β4^R^ mutant *T. cruzi* proteasomes in the presence and absence of ‘ES09 Series’ inhibitor DDD01012248 10](#_Toc182397729)

[Figure S5. Inhibition of trypsin-like activities of native and recombinant proteasomes 11](#_Toc182397730)

[Figure S6. Inhibition of caspase-like activities of native and recombinant proteasomes 12](#_Toc182397731)

[Figure S8. Inhibition of chymotrypsin-like activities of native and recombinant proteasomes for DDD01715999 and DDD01511935 14](#_Toc182397732)

[Figure S9. Native *T. cruzi* apo proteasome CryoEM data processing 15](#_Toc182397733)

[Figure S10. Recombinant *T. cruzi* apo proteasome CryoEM data processing 17](#_Toc182397734)

[Table S1. Identification of *T. cruzi* proteasome subunits in purified recombinant sample. Peptides listed in red were detected. 19](#_Toc182397735)

[Table S2. RMSD values (Å) for alignments of Cα of individual chains and percentage identity of respective 20S proteasomes 21](#_Toc182397736)

[Table S3. In-house proteasome series compound structures 23](#_Toc182397737)

[Table S4. Native, wild-type and mutant proteasome pIC_50_ values generated in chymotrypsin, trypsin and caspase activity assays 25](#_Toc182397738)

[Table S5. Wild-type and Δβ1Δβ2 mutant proteasome maximum % inhibition plateaus generated in chymotrypsin activity assays 27](#_Toc182397739)

[Table S6. Cryo-EM structure determination; microscope data collection parameters 28](#_Toc182397740)

[Table S7. Model refinement statistics 29](#_Toc182397741)

[DDD02091966 synthesis and NMR 30](#_Toc182397742)

## Figure S1. Expression and purification of recombinant *T. cruzi* proteasomes


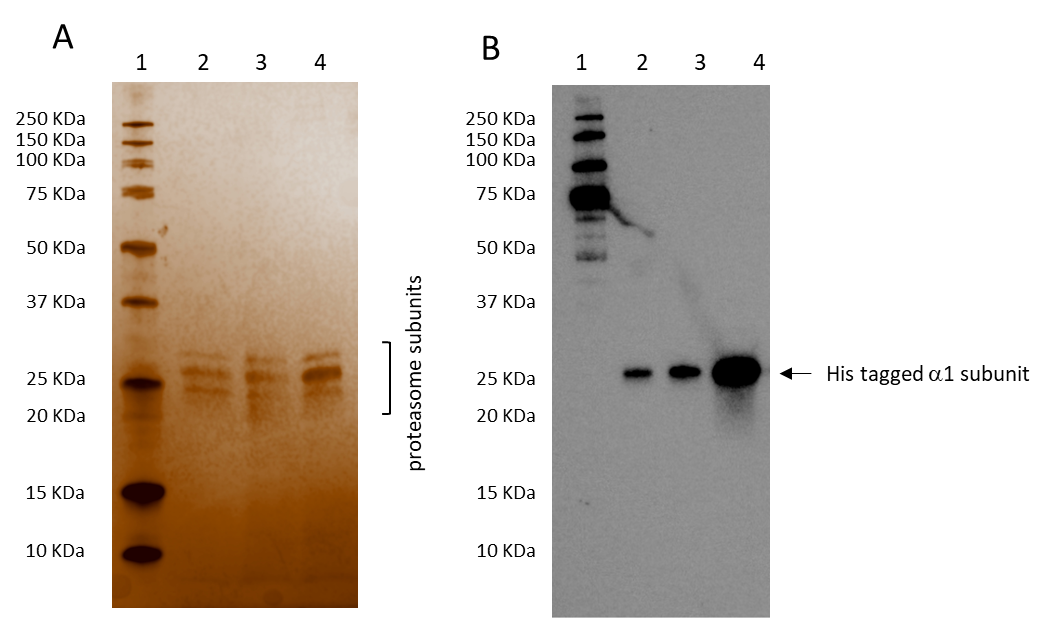


1. Silver stain gel of recombinant proteasomes (lane 2: Δβ1Δβ2, lane 3: β4^R^, lane 4: wild-type). B. Western blot of purified recombinant proteasomes using anti-His tag monoclonal antibody (lane 2: Δβ1Δβ2, lane 3: β4^R^, lane 4: wild-type).

## Figure S2. Final *T. cruzi* proteasome CryoEM maps with refined structures

a) Electron potential maps for the native *T. cruzi* apo proteasome. Upper image shows density for a well-defined region centred on β7 residue Arg 37, contoured at 1.5σ (water molecules shown as red spheres). Lower image shows density for the α3 subunit C-terminal helix contoured at 1.0σ, which became uninterpretable beyond residue 268.


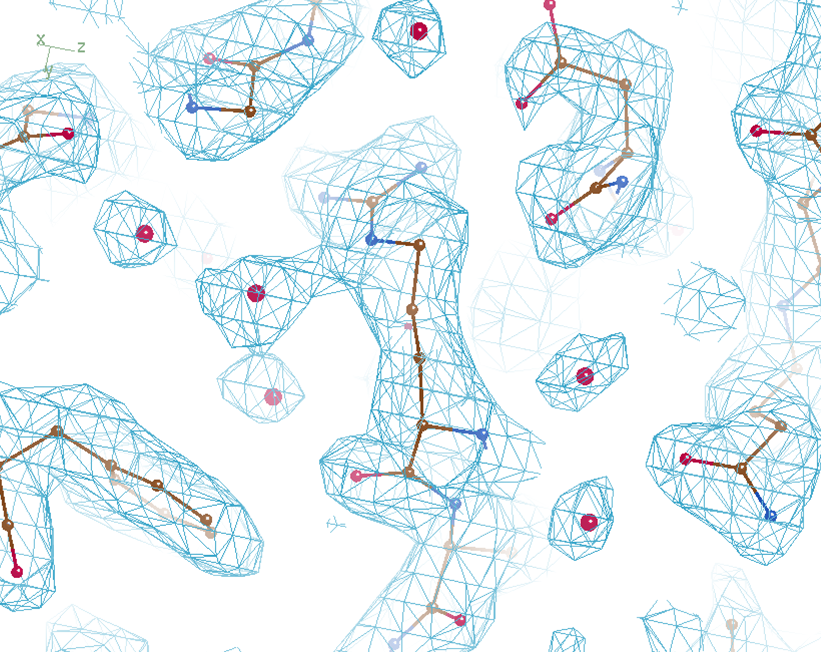


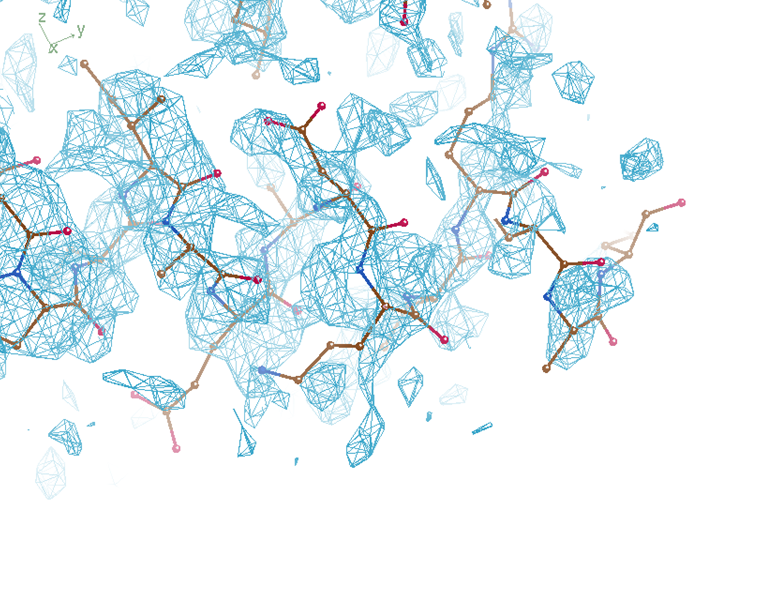


b) Electron potential maps for the recombinant *T. cruzi* apo proteasome. Upper image shows density for a well-defined region, centred on β7 residue Arg 37, contoured at 1.5σ (water molecules shown as red spheres). Middle image shows density for the α3 subunit C-terminal helix contoured at 1.0σ, which had considerably better density for the main chain atoms than that of the native map, allowing modelling of additional residues. Lower images show density for specific cysteine residues contoured at 1.5σ which appear to be oxidised.


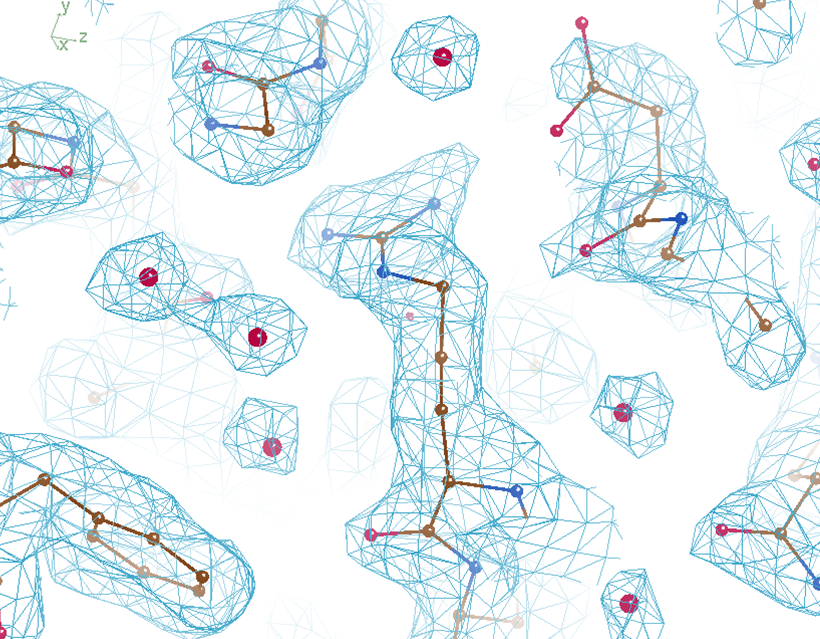


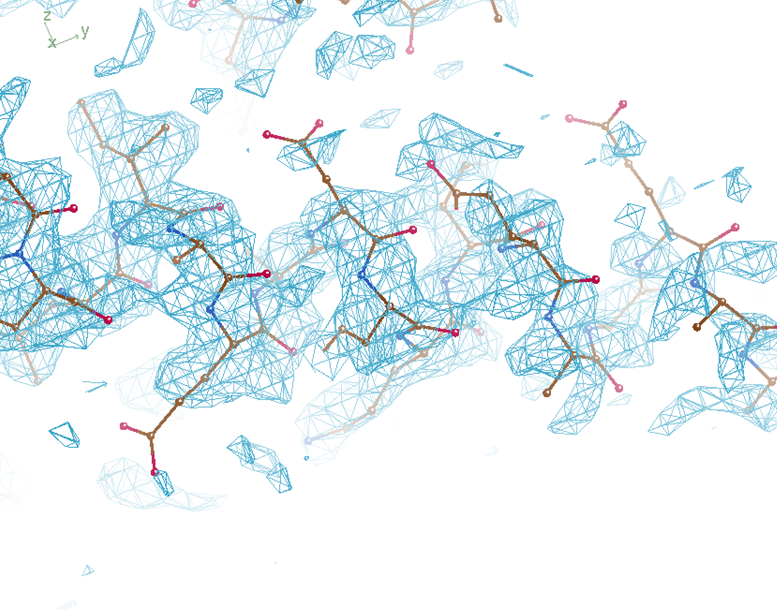


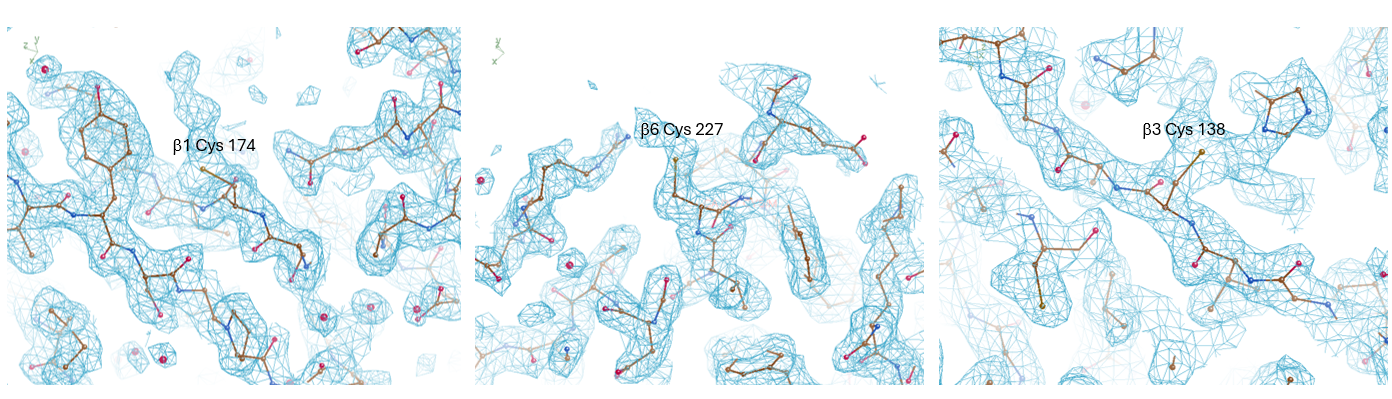


c) Cartoon representation of the refined structures, as coloured by B-factor (blue-lightblue-salmon-red) from 10 to 40 Å^2^ (blue to red).

| Native *T. cruzi* apo proteasome | Recombinant *T. cruzi* apo proteasome |
| --- | --- |
| 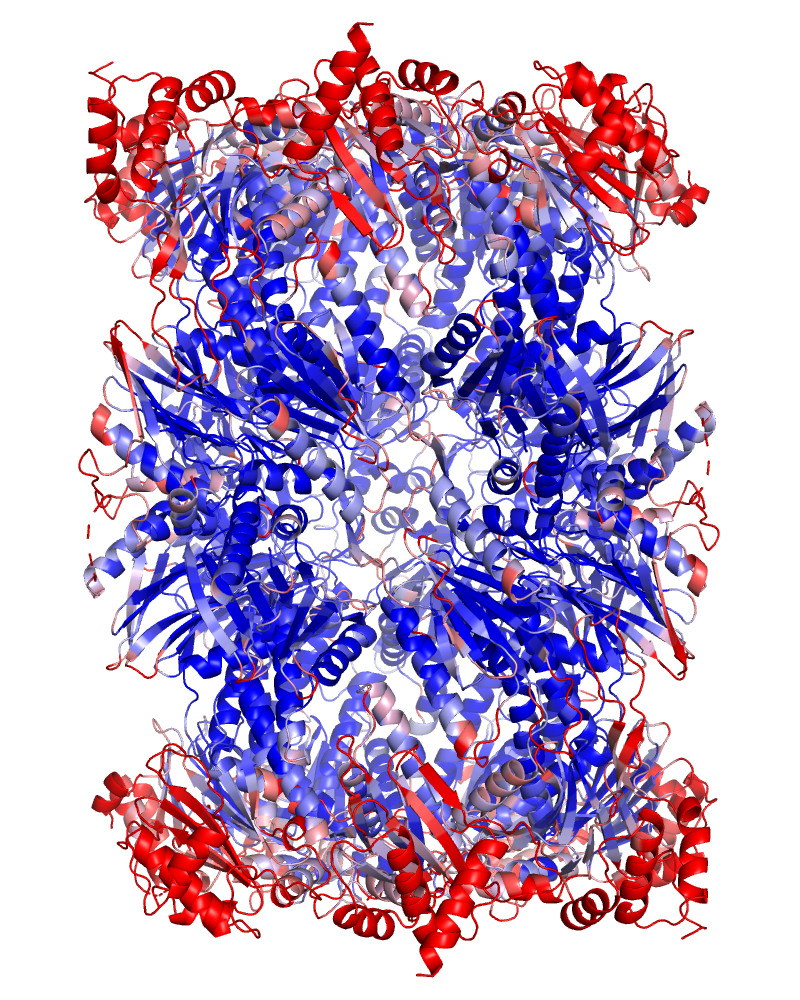 | 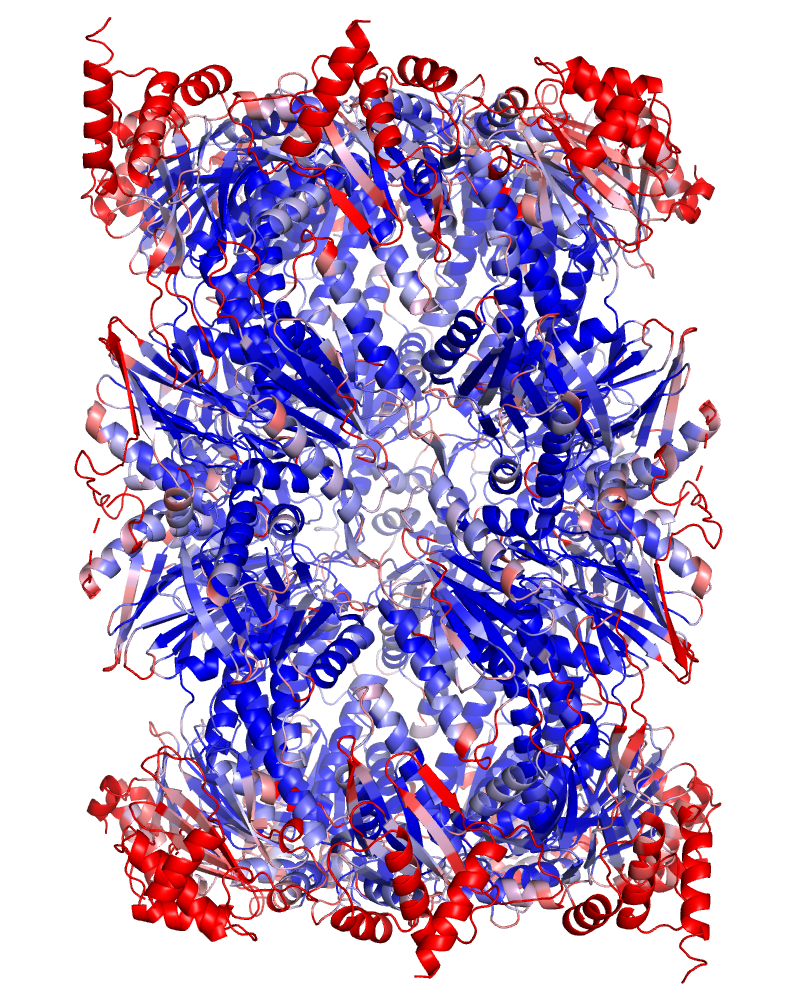 |

## Figure S3. Multiple sequence alignment of *T. cruzi*, *T. vaginalis*, *H. sapiens,* *L. tarentolae* and *P. falciparum* catalytic β1, β2 and β5 subunits

β1 subunits


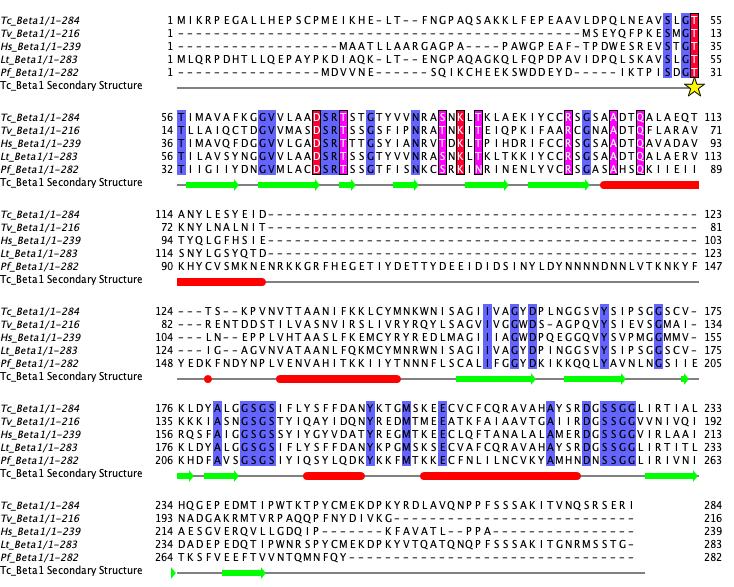


β2 subunits


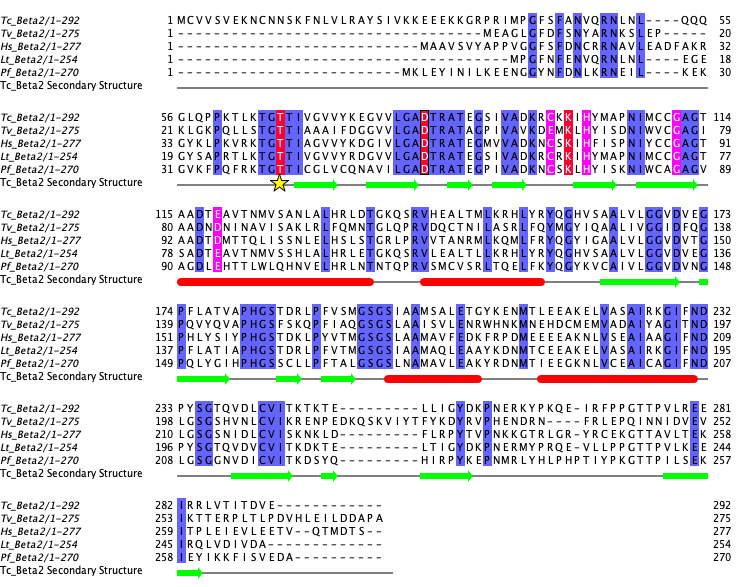


β5 subunits


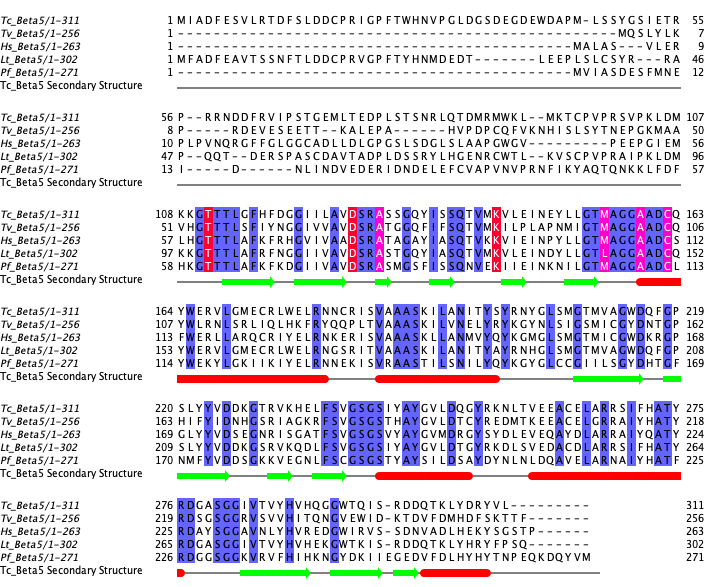


Catalytic residues coloured red, with residues involved in P1 specificity coloured hot pink. Residues mutated to Alanine for the *T. cruzi* Δβ1β2 mutant (Thr55 and Thr67 respectively) are highlighted with a yellow star. Residues coloured blue are identical between all species. Alignment produced using JalView^1^.

## Figure S4. Trypsin- and caspase-like activities of recombinant wild-type and β4^R^ mutant *T. cruzi* proteasomes in the presence and absence of ‘ES09 Series’ inhibitor DDD01012248

**
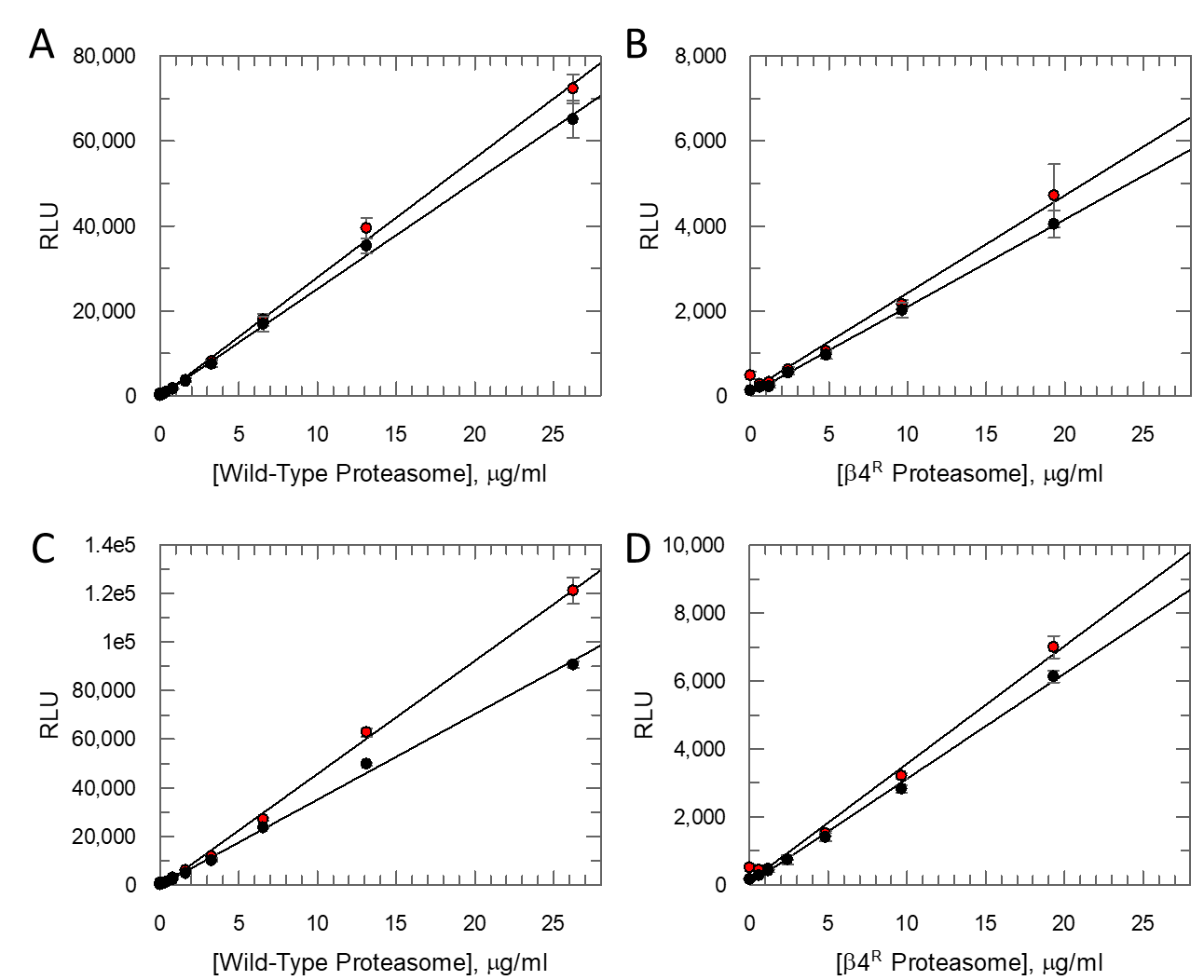
**

Trypsin activity measured for (A) recombinant wild-type and (B) recombinant β4^R^ mutant *T. cruzi* proteasomes; Caspase activity measured for (C) recombinant wild-type and (D) recombinant β4^R^ mutant *T. cruzi* proteasomes. In all graphs enzyme activities are plotted against *T. cruzi* proteasome concentration either in the presence (black circles) or absence (red circles) of 1 µM DDD01012248. Data are presented as mean RLU ± SD (*n* = 3 technical replicates). Solid lines are linear regression of the data. In all cases correlation coefficient > 0.99.

## Figure S5. Inhibition of trypsin-like activities of native and recombinant proteasomes

**
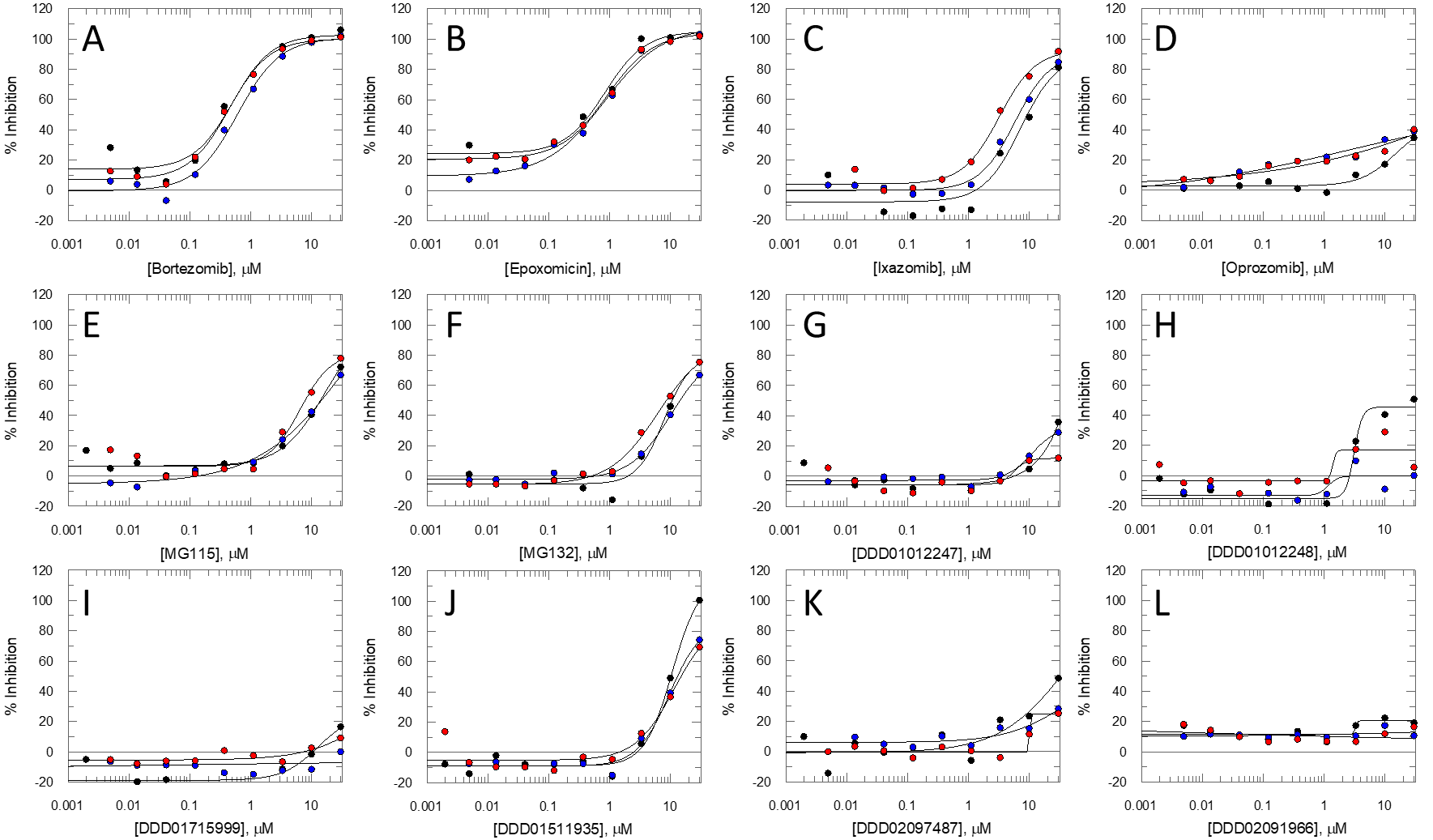
**

Panels A-L show representative trypsin assay dose response curves (*n* = 1) for ‘Proteasome Reference Panel’, ‘ES09 Series’, DDD02097487 and DDD02091966 against native (black), recombinant wild-type (blue) and recombinant β4^R^ mutant (red) *T. cruzi* proteasomes. Data were fitted to the 4-parameter logistic fit model (*equation 2* in Materials and Methods). Mean trypsin pIC_50_ data for these compounds, from independent biological replicates (*N* = 3), can be found in Table S4.

## Figure S6. Inhibition of caspase-like activities of native and recombinant proteasomes

**
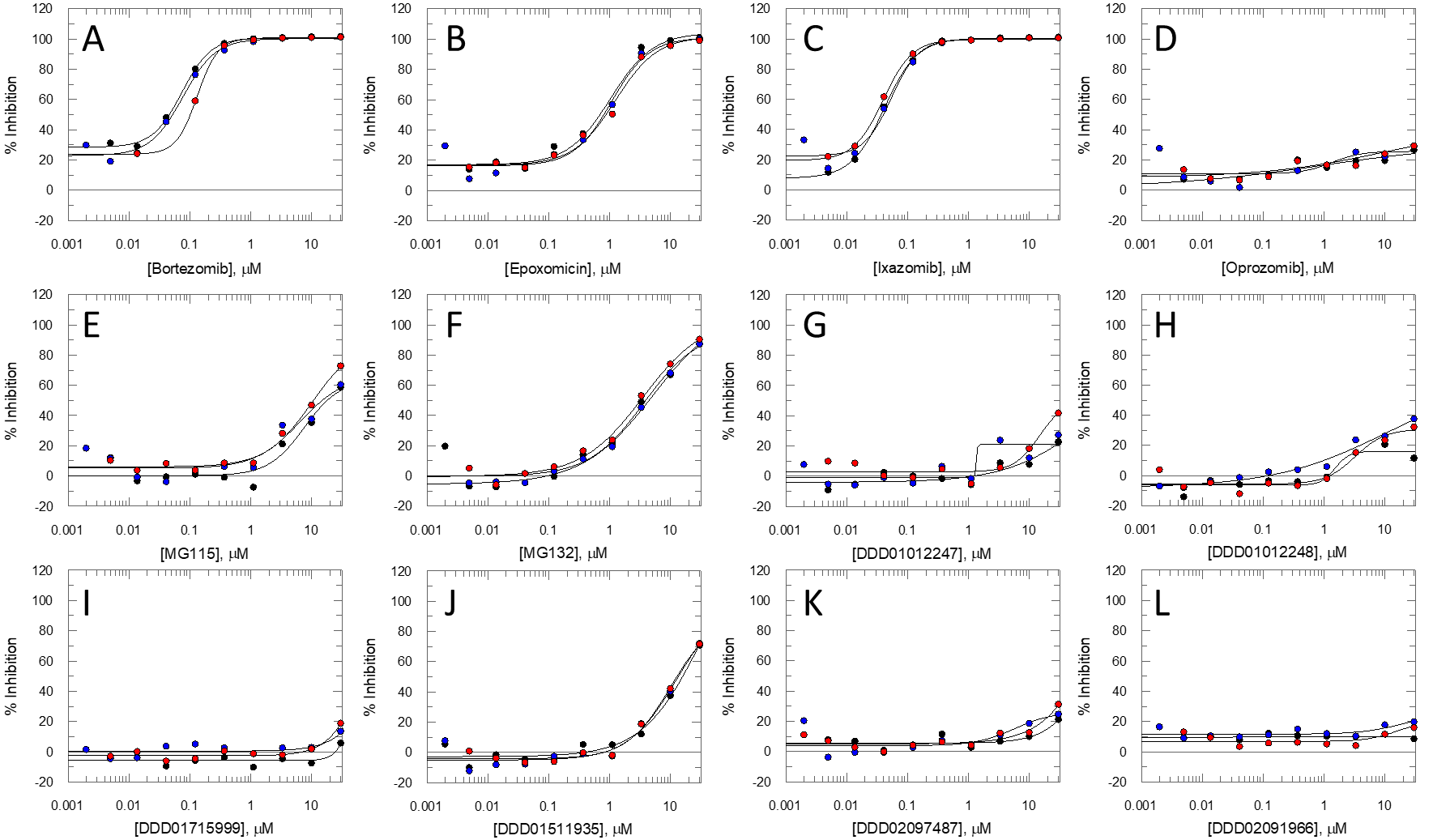
**

Panels A-L show representative caspase assay dose response curves (*n* = 1) for ‘Proteasome Reference Panel’, ‘ES09 Series’, DDD02097487 and DDD02091966 against native (black), recombinant wild-type (blue) and recombinant β4^R^ mutant (red) *T. cruzi* proteasomes. Data were fitted to the 4-parameter logistic fit model (*equation 2* in Materials and Methods). Mean caspase pIC_50_ data for these compounds, from independent biological replicates (*N* = 3), can be found in Table S4.**Figure S7.** **DDD02091966 does not inhibit human proteasome chymotrypsin activity.**


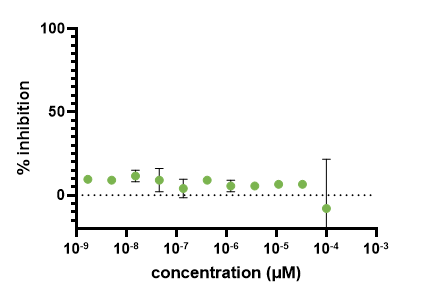


Dose response curve for DDD02091966 against the chymotrypsin activity of the human proteasome (*n* = 2).

## Figure S8. Inhibition of chymotrypsin-like activities of native and recombinant proteasomes for DDD01715999 and DDD01511935

Representative chymotrypsin assay dose response curves for (A) DDD01715999 and (B) DDD01511935 against native (black), recombinant wild-type (blue), recombinant β4^R^ mutant (red) and recombinant Δβ1Δβ2 mutant (green) *T. cruzi* proteasomes. Data are presented as mean % inhibition ± SD (*n* = 3 technical replicates). Data for independent replicates are shown in **Figure 9M** with mean pIC_50_ data reported in Table S4. Data were fitted to a 4-parameter logistic fit model (*equation 2* in Materials and Methods) or, for DDD01511935 (black and blue curves) a biphasic fit model (*equation 3* in Materials and Methods).

## Figure S9. Native *T. cruzi* apo proteasome CryoEM data processing

a) Final selected classes after 2D classification (214,900 particles).

Highlighted classes are sample impurities (not proteasome particles).


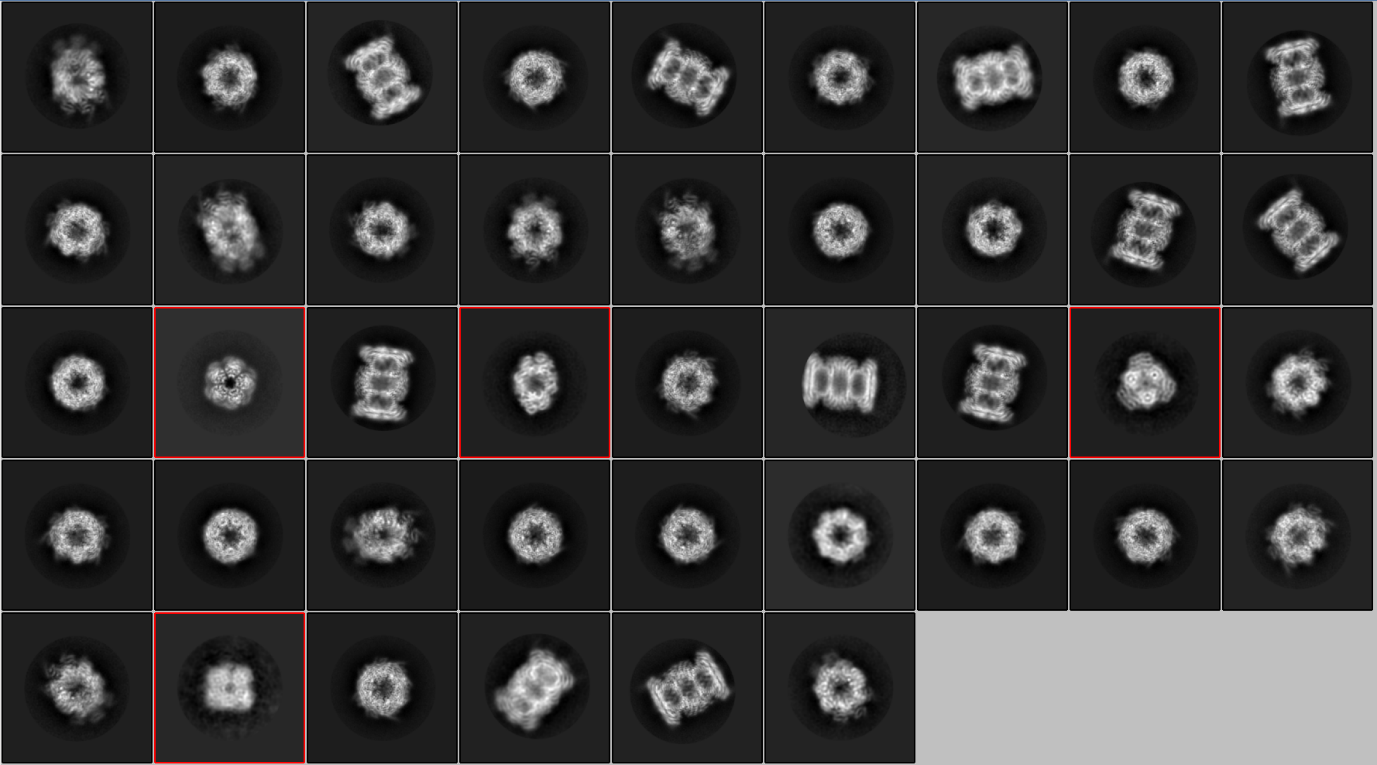


b) Final classes after 3D classification with C2 symmetry, selected class highlighted (50,663 particles).


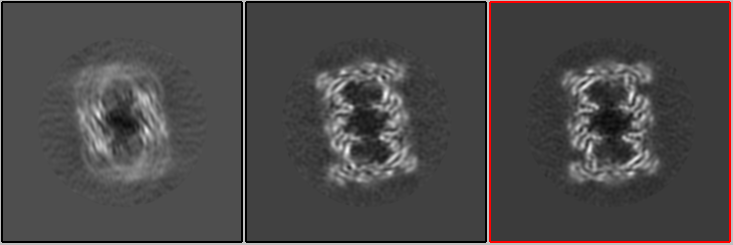


c) Angular distribution of particle views for final Refine3D map.


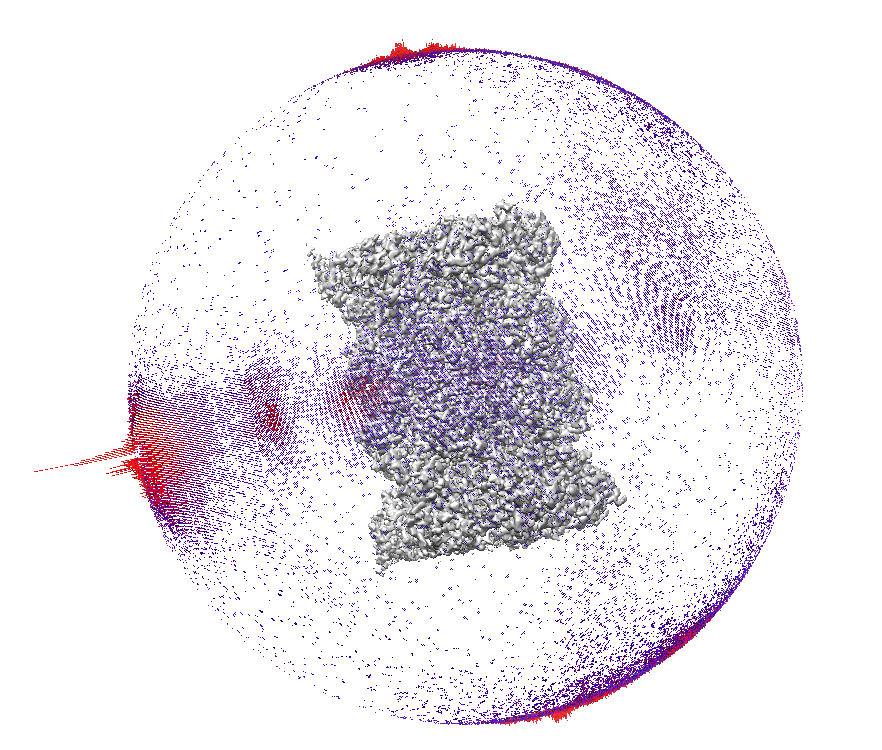


d) Fourier shell correlation (FSC) plot.


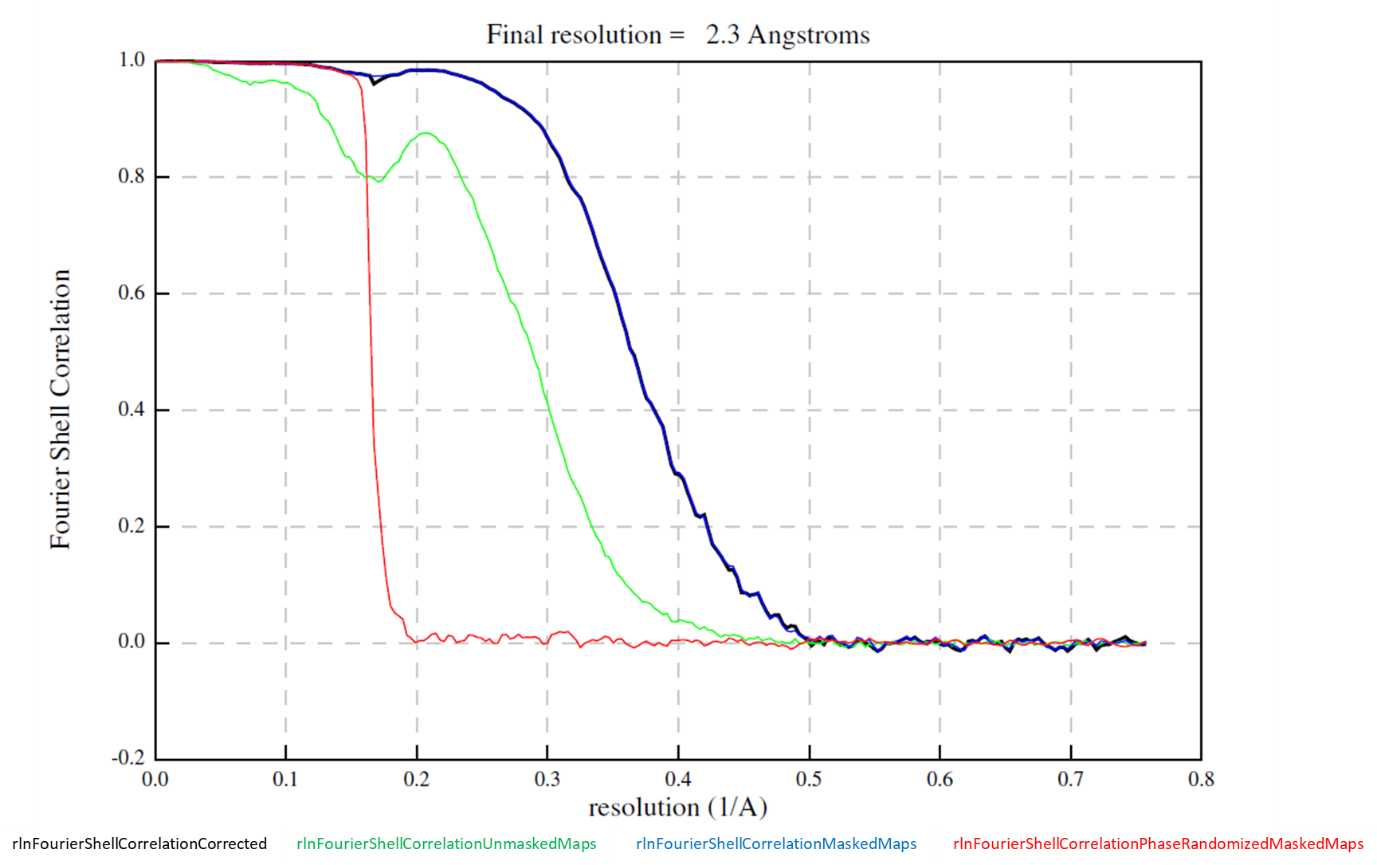


## Figure S10. Recombinant *T. cruzi* apo proteasome CryoEM data processing

a) Final selected classes after 2D classification (113,240 particles).


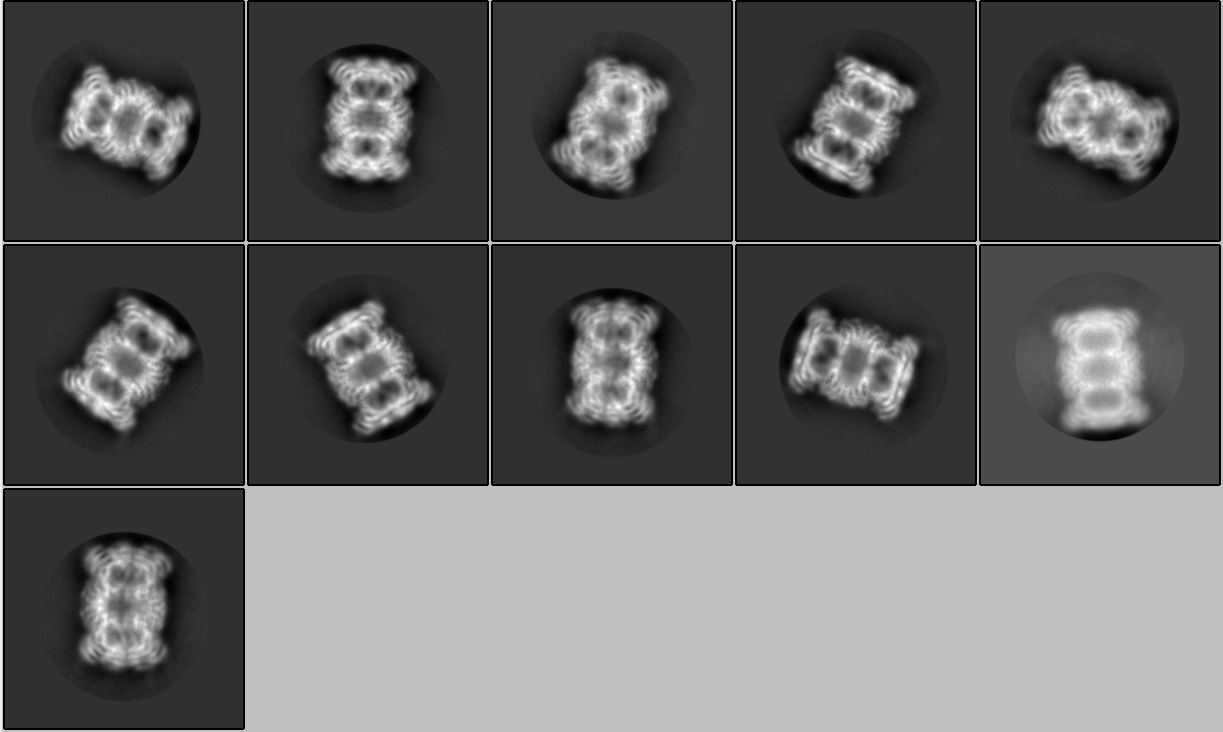


b) Final classes after 3D classification with C2 symmetry, selected class highlighted (92,013 particles).


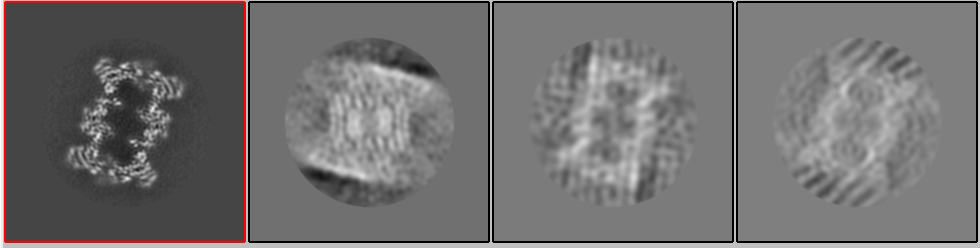


c) Angular distribution of particle views for final Refine3D map.


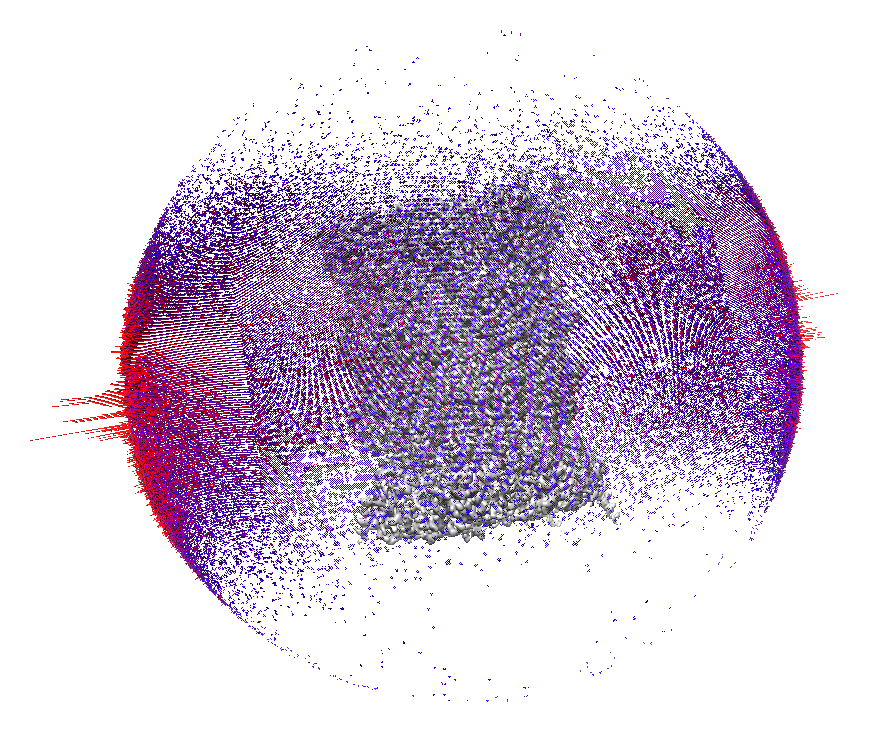


d) Fourier shell correlation (FSC) plot.


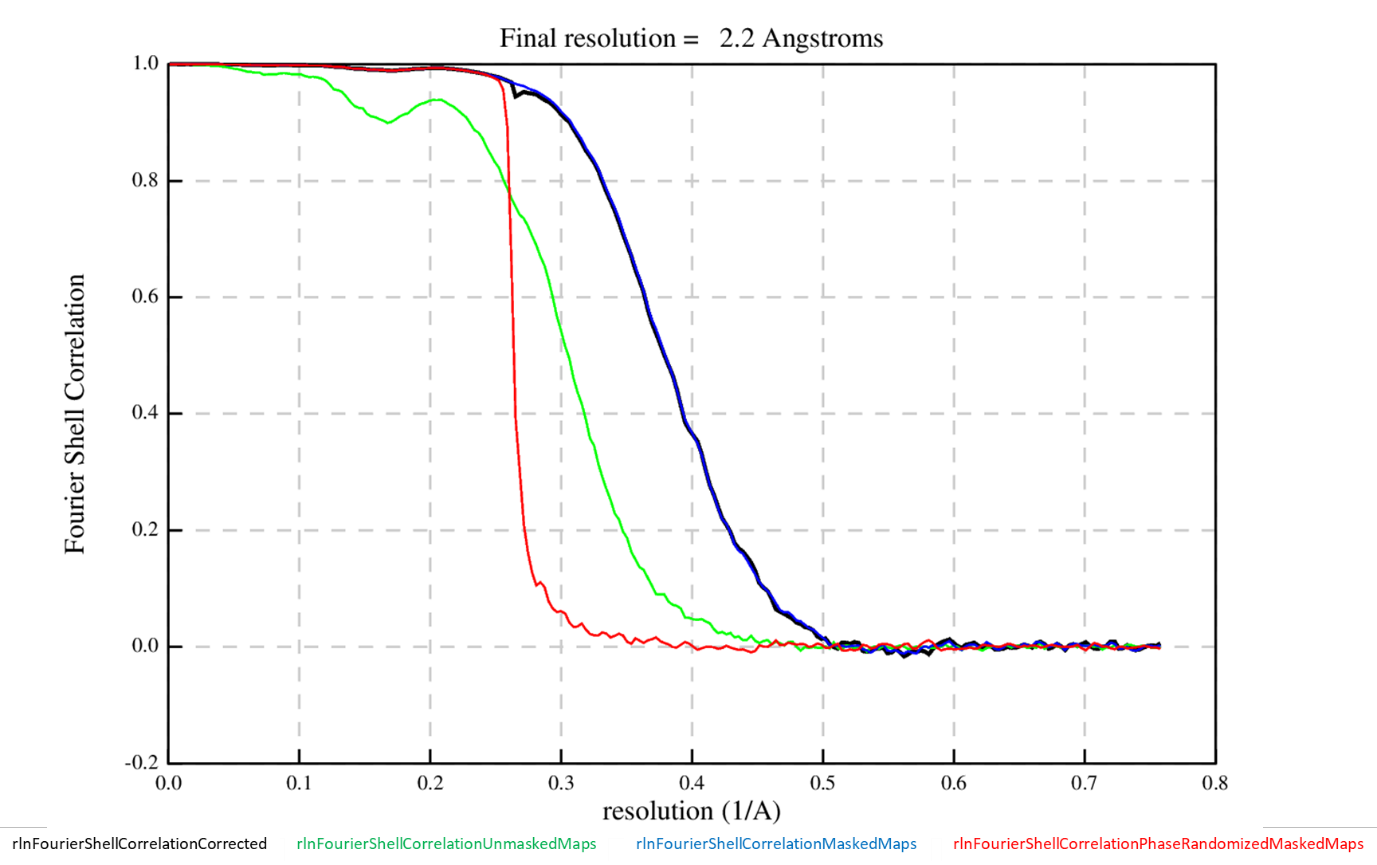


Supplementary Tables

## Table S1. Identification of *T. cruzi* proteasome subunits in purified recombinant sample. Peptides listed in red were detected.

| Subunit / TriTrypDB ID | Sequence | Coverage | Score* | Mass |
| --- | --- | --- | --- | --- |
| α1  TCSYLVIO_005678-t26_1 | MSRTGFDKYITVFSPEGSLYQVEYAFKAVTYAGLLTVAIRCKDAVLVFTQHNVPDRLMRPDTITSLHNINKDIGTCITGRAPDGKSLVQKARQEASEYKYRYGSPIPVSVLAKRVADMAQVRTQHAGLRLMGVVMTFLGMEQNDADGSWVPQIYCVDPAGWCGSYHACAIGKKQIEANAFLEKKQKNAPFHTLSQKDAAMIALAALQSALGASLKATDVEVGRCSVEDHCFRRVPDEDVEEWLTALAEAD | 62% | 2235 | 28,050 |
| α2  TCSYLVIO_009369-t26_1 | MSESAYGLTTFSPSGRLVQIEYATTAASKGTTALGVKAMDGVVIAAEKKTTSPLAASLTVQKVFVLDDHVGCTYSGIGPDCRVLVDAARKACQKYRLTYHEPMPVSQLVRHISSLYQEFTQSGGVRPFGCSLLVAGADSQGNHLYQVDPSGTFWAWKATSIGKGSTDAKTFLEKRYTNEMEIEDAVHTALLTLKEGFDGTMTAENTQVGRVSEGKFELFTVDQLKDYLDQGRIPAHWRPLLVDPSSVPSLMHSLSIL | 43% | 5398 | 27,901 |
| α3  TCSYLVIO_010341-t26_1 | MSSRYDSRTTTFSPEGRLYQVEYAEEAISQAGTVIGILTTGGVVLGAEKGVQNSLFDSENMEDKNISGEKMYKIASHIGCSVAGVTSDAYALLNYARLSANRHHYTYQEPMAAEDLCRLLCDEKQLYTQYGGVRPFGVSFLLAGWDRHHGYQLYHTDTSGNYNAWRAYAIGQNDQVAQSLLKRDWKPELTLDEGIVLCLRVLGKTMDTVKLSAERLEVAVLHKVPAPATQKLLEPYGVLPKTVPEFKILRETDLKPLIAEADRQREAEEAAEAEKEKKKEQKLTSS | 44% | 2216 | 31,936 |
| α4  TCSYLVIO_001707-t26_1 | MSYDRAITVFSPDGHLFQVEYAQEAVRKGLCAVGVRGKDSIIFAVEKKSVQKLQDSRTIRKIYKLDEHIYLAFAGLSADARVLVNHAQLECQRFRLNYEDAVDVDLLVRYVAKVQQKSTQSSGSRPYGVSTIIGGFNENGQPHLWKTDPSGMSSAWRAVAIGRNDKTVLEFMEKSYQENMTRDQCVHFAIKALLEAVESGSKNIELVVLENKKALYMGDDELRKFVVEVEKEREEEAARKRRLAEEE | 75% | 5599 | 28,050 |
| α5  TCSYLVIO_009373-t26_1 | MFSSKTEYDRGVNTFSPEGRIFQIEYAIEAIKLGSTSLGIQTPDAVVIAAEKRVPSVLVDPSSMNKILEIDYHMGTVLSGMVADARILVEHARVEAQNHRFTYDEPMRVESCALATCDLSVRFGESGGRKKLMSRPFGVSLLIAGVDENGPQLWQTDPSGTYTRYDAQAIGAGAEAAQTVFNEIYHRNMTVEEAETLAVRILKQVMEEELTKSNIEIAIVPASTGKLEVYDQTKIQRIIDRLTEE | 67% | 5068 | 27,118 |
| α6  TCSYLVIO_004937-t26_1 | MFKNQYDTNTTTWSPTGRLFQVEYANEAVNNGSAAVGTKNKDFVVLTALKRSPVAQFSSYQEKVFKLDDHVGMAIAGLVADGRMLARFVRTECMNYRYMHDSDNPLPLIAEIVGEKYQRHIQFAGKRPFGVGLLIAGYDSTGPHLYHTVPSGDVFDFKATAMGLRSQAARTYLEKHFESFPDCGLDELIMHALKALAAATSGGAELNIKNTTIAIVGKGTPFMVLTEEAARKYLDGFKMRPEDIPPAPEAEEEETLQERSLDVEE | 89% | 18,387 | 29,347 |
| α7  TCSYLVIO_003527-t26_1 | MSGTEHDQSTDIFSADGRVFQVEYACKAVDNGSTAVAACCTDGVVVAVEKILTSRMLEEGSNDRIHAVDRQAGVCICGMLPDGRAVVSRARAEAENSRDVFATPIPGSVLASRIGEFMHVYTTHYAYRPFGCSVIIASYADDGPQLFVSDPSGTVAGYYGIALGKGKTVAKTELEKLNFKSITCDEAVVKLTKILHDVHDKSKDKLYELEVAWVCNKSNCVFQHVPNDMIPKPPASQ | 38% | 1018 | 30,725 |
| β1  TCSYLVIO_006999-t26_1 | **MIKRPEGALLHEPSCPMEIKHELTFNGPAQSAKKLFEPEAAVLDPQLNEAVSLG**TTIMAVAFKGGVVLAADSRTSTGTYVVNRASNKLTKLAEKIYCCRSGSAADTQALAEQTANYLESYEIDTSKPVNVTTAANIFKKLCYMNKWNISAGIIVAGYDPLNGGSVYSIPSGGSCVKLDYALGGSGSIFLYSFFDANYKTGMSKEECVCFCQRAVAHAYSRDGSSGGLIRTIALHQGEPEDMTIPWTKTPYCMEKDPKYRDLAVQNPPFSSSAKITVNQSRSERI | 45% | 1018 | 30,725 |
| β2  TCSYLVIO_005774-t26_1 | **MCVVSVEKNCNNSKFNLVLRAYSIVKKEEEKKGRPRIMPGFSFANVQRNLNLQQQGLQPPKTLKTG**TTIVGVVYKEGVVLGADTRATEGSIVADKRCKKIHYMAPNIMCCGAGTAADTEAVTNMVSANLALHRLDTGKQSRVHEALTMLKRHLYRYQGHVSAALVLGGVDVEGPFLATVAPHGSTDRLPFVSMGSGSIAAMSALETGYKENMTLEEAKELVASAIRKGIFNDPYSGTQVDLCVITKTKTELLIGYDKPNERKYPKQEIRFPPGTTPVLREEIRRLVTITDVE | 58% | 1640 | 31,992 |
| β3  TCSYLVIO_001736-t26_1 | MSILTYSGGSCLAMAGDGCFVIVSDNRLGEQLKTISMEVPKLHVINESIVLGLTGLRTDQQTFSEKVRFRNELYKLREEREIGGKAFAALVSSMLYEARFGPWFVEPVIASIDKRTGEVYLCAMDLIGAPCEPEDYVCAGTCAESLHGMCEALWRPGLGPEELFEVAAQAMLSACDRDSLSGYGAVAAIVTRDKMTTRLINGRKD | 43% | 2132 | 22,387 |
| β4  TCSYLVIO_007432-t26_1 | MSETTIAFRCNGFVLVAAAGLNAFYYIKIMDTEDKVTQLDSHKVVACAGENGPRVNFVEYIKCNMALKRMREHGRVIRTSAAASFMRNALAGALRSRDGAYLVNCLLAGYDVAASSDDDIATGPHLYYMDYLGTMQEVPYGCHGYGASFVIAMLDRLWRPDLTAQEAVDLMQKCCDEVKKRVVISNDKFICKAVTENGVEIVNTVS | 31% | 831 | 22,704 |
| β5  TCSYLVIO_004939-t26_1 | **MIADFESVLRTDFSLDDCPRIGPFTWHNVPGLDGSDEGDEWDAPMLSSYGSIETRPRRNDDFRVIPSTGEMLTEDPLSTSNRLQTDMRMWKLMKTCPVPRSVPKLDMKKG**TTTLGFHFDGGIILAVDSRASSGQYISSQTVMKVLEINEYLLGTMAGGAADCQYWERVLGMECRLWELRNNCRISVAAASKILANITYSYRNYGLSMGTMVAGWDQFGPSLYYVDDKGTRVKHELFSVGSGSIYAYGVLDQGYRKNLTVEEACELARRSIFHATYRDGASGGIVTVYHVHQGGWTQISRDDQTKLYDRYVL | 42% | 1244 | 34,916 |
| β6  TCSYLVIO_002482-t26_1 | MIEDHMEYGHHFPRKLADSTLSLPRQGVKEQQWSPYADNGGTIAAIAGKNYVILGGDTRLNGDFCIHTRDDRTKLFQLTEHTFLASTGMQADRLQLQQMLKYRIQWYQYNNGGKLPSTKAIAKLTSTMLYQRRFFPYYTFNMVVGLDEKGAGVCYSYDPVGSTEPFRYGTSGSASSFVEPLMDCLLTRQHMVQQAPAELSMTETLEMLKNAFTGAAERDIFTGDAVCFHIITADGIRSELFELRND | 56% | 1575 | 27,784 |
| β7  TCSYLVIO_003686-t26_1 | MASGGSVIGVKYNGGVLLACDTLLSYGSLAKWPNIPRMKLVGAYTVMCATGDYADFQEMTTMIENHVNRQQMYGGGALTPNEVFCYLQRHVYHKRSQFEPCLCRFVVAGCHGGEPFLGGVDDVGTRWTDDCVAAGYGAYVALPLLRQALEKPGGLTREEAIRVIKDCLRVLFYRECRAINKFQIADATSDMVSIGEPFEVETNWEYDGFCFEKTAIIR | 22% | 207 | 24,182 |

*score is the sum of ion scores, where ion score is -10*Log(P), where P is the probability that the observed match is a random event.

Predicted pro-peptide of β1, β2 and β5 subunits highlighted in bold and underlined.

## Table S2. RMSD values (Å) for alignments of Cα of individual chains and percentage identity of respective 20S proteasomes

| Subunit | *L. tarentolae (6QM7)* | | *H. sapiens (6RGQ)* | | *T. vaginalis (8OIX)* | | *P. falciparum (6MUW)* | |
| --- | --- | --- | --- | --- | --- | --- | --- | --- |
|  | RMSD | % sequence identity | RMSD | % sequence identity | RMSD | % sequence identity | RMSD | % sequence identity |
| α1 | 0.701 over 244 pairs | 70.8 | 1.961 over 233 pairs | 44.86 | 1.975 over 236 pairs | 35.68 | 2.94 over 237 pairs | 34.29 |
| α2 | 0.625 over 228 pairs | 77.92 | 1.031over 227 pairs | 52.16 | 2.149 over 224 pairs | 48.48 | 1.279 over 227 pairs | 46.12 |
| α3 | 0.992 over 271 pairs | 70.88 | 3.475 over 245 pairs | 51.37 | 4.219 over 235 pairs | 50 | 5.740 over 241 pairs | 50 |
| α4 | 0.890 over 234 pairs | 77.73 | 1.391 over 229 pairs | 55.51 | 1.829 over 229 pairs | 46.98 | 1.55 over 233 pairs | 51.04 |
| α5 | 0.734 over 227 pairs | 77.87 | 1.356 over 225 pairs | 53.53 | 1.310 over 227 pairs | 48.98 | 2.071 over 227 pairs | 50.2 |
| α6 | 0.859 over 221 pairs | 73.86 | 1.061 over 221 pairs | 42.75 | 1.482 over 218 pairs | 42.92 | 1.435 over 220 pairs | 39.37 |
| α7 | 0.638 over 227 pairs | 79.66 | 0.919 over 226 pairs | 47.26 | 1.438 over 224 pairs | 37.18 | 2.343 over 223 pairs | 30.8 |
|  |  |  |  |  |  |  |  |  |
| β1 | 0.744 over 228 pairs | 80.35 **(75.97)** | 7.610 over 202 pairs | 49.76 **(47.66)** | 1.670 over 201 pairs | 37.62 (**36.45)** | 2.208 over 194 pairs | 36.36 **(34.64)** |
| β2 | 0.740 over 219 pairs | 86.67 (**84.65)** | 1.004 over 219 pairs | 57.08 **(53.96)** | 2.201 over 218 pairs | 43.44 **(43.37)** | 2.560 over 218 pairs | 50.88 **(47.94)** |
| β3 | 0.547 over 204 pairs | 84.39 | 0.908 over 204 pairs | 43.9 | 0.995 over 204 pairs | 49.27 | 0.995 over 202 pairs | 40.98 |
| β4 | 0.668 over 206 pairs | 69.9 | 1.175 over 195 pairs | 38.46 | 1.499 over 191 pairs | 34.55 | 2.453 over 192 pairs | 34.02 |
| β5 | 0.555 over 201 pairs | 86.57 **(73.91)** | 0.699 over 200 pairs | 61.19 (**53.70)** | 1.396 over 198 pairs | 53.23 **(45.24)** | 2.934 over 188 pairs | 55.72 **(49.19)** |
| β6 | 0.689 over 208 pairs | 76.02 | 1.842 over 206 pairs | 40.42 | 2.141 over 206 pairs | 32.59 | 1.186 over 206 pairs | 35 |
| β7 | 0.841 over 216 pairs | 69.27 | 1.470 over 206 pairs | 41.31 | 2.089 over 203 pairs | 27.4 | 2.110 over 197 pairs | 31.02 |
|  |  |  |  |  |  |  |  |  |
| overall | 1.97 over 6256 pairs | 77.28 | 3.048 over 6076 | 48.54 | 4.301 over 6028 | 42.02 | 3.041 over 6010 | 41.04 |

RMSD values calculated for the Cα atoms using *T. cruzi* recombinant structure as the fixed model with Matchmaker in Chimera. Percentage sequence identity calculated using Clustal Omega on the EBI server. Values in parentheses are for the pre-digested subunits.

## Table S3. In-house proteasome series compound structures

| Structure | Compound ID | Cpd Nr. in Wyllie et al.^2^ | Cpd Nr. in Thomas et al.^3^ | Cpd Nr. in Thomas et al.^4^ |
| --- | --- | --- | --- | --- |
| \|  Fc1ccc(cc1-cn2nc(cnc2n1)N1CCOCC1)NC(=O)N1CCCC1 \| \| --- \| | DDD01012247 | 7a | 23 |  |
| \| Fc1ccc(cc1-c1cn2nc(cnc2n1)-c1ccccc1)NC(=O)N1CCCC1 \| \| --- \| | DDD01012248 | 7 |  |  |
| \| (cnc2n1)N1CCOCC1)NC(=O)N1CC(F)(F)C1 \| \| --- \| | DDD01511935 (*) |  |  |  |
| \| @H]1C O)-c1cc(ccc1F)NC(=O)N1CC[C@@H](F)C1 \| \| --- \| | DDD01715999 |  | close analog of 39 |  |
|  | DDD02097487 |  |  | 26 |
|  | DDD02091966 | Not previously reported, see supplementary methods. | | |

Green background: ES09 series compounds, (*): previously reported in patent WO2017025416

## Table S4. Native, wild-type and mutant proteasome pIC_50_ values generated in chymotrypsin, trypsin and caspase activity assays

| **Compound Panel** | **Compound ID** | **Chymotrypsin Assay** | | | | **Trypsin Assay** | | | **Caspase Assay** | | |
| --- | --- | --- | --- | --- | --- | --- | --- | --- | --- | --- | --- |
|  |  | **Native** | **Wild-type** | **β4^R^ mutant** | **Δβ1Δβ2** **mutant** | **Native** | **Wild-type** | **β4^R^ mutant** | **Native** | **Wild-type** | **β4^R^ mutant** |
| **Proteasome Reference Panel** | **Bortezomib** | 8.32 ± 0.16^a^ | 8.29 ± 0.32^a^ | 8.18 ± 0.28^a^ | 8.16 ± 0.48 | 6.27 ± 0.02 | 6.21 ± 0.03 | 6.32 ± 0.04 | 7.14 ± 0.01 | 7.14 ± 0.05 | 7.03 ± 0.13 |
|  | **Epoxomicin** | 7.50 ± 0.12 | 7.40 ± 0.17 | 7.33 ± 0.15 | 7.44 ± 0.03 | 6.08 ± 0.00 | 6.09 ± 0.04 | 6.11 ± 0.06 | 5.99 ± 0.01 | 5.97 ± 0.03 | 5.88 ± 0.03 |
|  | **Ixazomib** | 7.45 ± 0.18^a^ | 7.71 ± 0.14^a^ | 7.88 ± 0.25^a^ | 7.64 ± 0.20 | 5.06 ± 0.07 | 5.16 ± 0.08 | 5.53 ± 0.03 | 7.35 ± 0.03 | 7.26 ± 0.02 | 7.33 ± 0.08 |
|  | **Oprozomib** | 7.05 ± 0.17 | 7.03 ± 0.17 | 7.13 ± 0.12 | 7.02 ± 0.12 | <4.52 | <4.52 | <4.52 | <4.52 | <4.52 | <4.52 |
|  | **MG115** | 6.04 ± 0.08 | 6.07 ± 0.13 | 6.24 ± 0.05 | 6.38 ± 0.16 | 4.89 ± 0.16 | 4.97 ± 0.09 | 5.24 ± 0.05 | 4.79 ± 0.11 | 4.77 ± 0.07 | 5.03 ± 0.04 |
|  | **MG132** | 6.92 ± 0.23^a^ | 6.94 ± 0.28^a^ | 7.22 ± 0.27^a^ | 7.04 ± 0.31 | 5.05 ± 0.26 | 5.08 ± 0.32 | 5.21 ± 0.11 | 5.40 ± 0.05 | 5.48 ± 0.15 | 5.51 ± 0.11 |
| **ES09 Series** | **DDD01012247** | 7.92 ± 0.16 | 7.79 ± 0.19 | 5.16 ± 0.29 | 7.71 ± 0.19 | <4.52 | <4.52 | <4.52 | <4.52 | <4.52 | <4.52 |
|  | **DDD01012248** | 7.51 ± 0.11 | 7.28 ± 0.23 | 5.70 ± 0.22 | 7.38 ± 0.18 | <4.52^c^ | <4.52 | <4.52 | <4.52^d^ | <4.52 | <4.52 |
|  | **DDD01715999** | 8.18 ± 0.34 | 7.75 ± 0.22 | 5.08 ± 0.27 | 7.82 ± 0.21 | <4.52 | <4.52 | <4.52 | <4.52 | <4.52 | <4.52 |
|  | **DDD01511935** | 7.28 ± 0.26^a^ | 7.09 ± 0.18^a^ | 5.10 ± 0.08 | 6.82 ± 0.17 | 4.97 ± 0.07 | 4.94 ± 0.08 | 4.92 ± 0.01 | 4.79 ± 0.08 | 4.91 ± 0.11 | 4.97 ± 0.02 |
|  | **DDD02097487** | 6.92 ± 0.17 | 6.75 ± 0.08 | <4.52 | 6.81 ± 0.12 | <4.52 | <4.52 | <4.52 | <4.52 | <4.52 | <4.52 |
|  | **DDD02091966** | 7.42 ± 0.42 | 7.46 ± 0.29^b^ | <4.52 | 7.34 ± 0.18 | <4.52 | <4.52 | <4.52 | <4.52 | <4.52 | <4.52 |

Data presented are mean pIC_50_ ± SD from *N* = 3 independent biological replicates.

^a^ Data generated from biphasic curve fits (*equations 3* or *4*), with pIC_50_ values for the most potent curve reported (corresponding to inhibition of the chymotrypsin site).

^b^ *N* = 2

^c^ Two of the three replicates tested returned pIC_50_ <4.52, the third replicate returned pIC_50_ of 5.50.

^d^ Two of the three replicates tested returned pIC_50_ <4.52, the third replicate returned pIC_50_ of 5.80.

## Table S5. Wild-type and Δβ1Δβ2 mutant proteasome maximum % inhibition plateaus generated in chymotrypsin activity assays

| **Compound Panel** | **Compound ID** | **Chymotrypsin Assay** | |
| --- | --- | --- | --- |
|  |  | **Wild-type** | **Δβ1Δβ2 mutant** |
| **Proteasome Reference Panel** | **Bortezomib** | 57.6 ± 13.7 ^a^ | 100.4 ± 0.1 |
|  | **Epoxomicin** | 96.1 ± 3.0 | 100.1 ± 0.5 |
|  | **Ixazomib** | 52.7 ± 21.9 ^a^ | 100.5 ± 0.5 |
|  | **Oprozomib** | 79.0 ± 2.3 | 100.2 ± 0.3 |
|  | **MG115** | 93.3 ± 2.9 | 99.4 ± 1.0 |
|  | **MG132** | 66.1 ± 6.6 ^a^ | 100.6 ± 0.8 |
| **ES09 Series** | **DDD01012247** | 68.9 ± 3.5 | 97.6 ± 0.2 |
|  | **DDD01012248** | 67.7 ± 4.0 | 95.0 ± 2.2 |
|  | **DDD01715999** | 65.1 ± 2.2 | 97.8 ± 0.4 |
|  | **DDD01511935** | 57.9 ± 5.4 ^a^ | 100.3 ± 0.6 |
|  | **DDD02097487** | 48.2 ± 3.6 | 69.3 ± 0.4 |
|  | **DDD02091966** | 51.6 ± 1.8 | 74.1 ± 0.7 |

Data presented are mean maximum % inhibition plateau ± SD from *N* = 3 independent biological replicates (except *N* = 2 for DDD02091966 against the wild-type proteasome).

^a^ Maximum % inhibition plateaus reported for these compounds are the mid-point plateau from biphasic curve fits (*equations 3* or *4*).

## Table S6. Cryo-EM structure determination; microscope data collection parameters

| **Hardware** | |
| --- | --- |
| Microscope | Krios |
| Detector (mode) | K3 (Counting Super Resolution) |
| Accelerating voltage (kV) | 300 |
| Detector Pixel size (Å) | 0.66 |
|  | |
| **Illumination parameters** | |
| Gun lens | 5 |
| Spot size | 6 |
| Illuminated area (µm) | 0.6 |
|  | |
| **Dose** | |
| Nominal Magnification | 130,000 |
| Square pixel (Å^2^) | 0.66 x 0.66 |
| Exposure time (s) | Native 2, Recombinant 1.41 |
| Number of Fractions | 40 |
| Total dose (e^-^/Å^2^) | Native 57.1, Recombinant 42 |
|  | |
| **Apertures (μm)** | |
| C1 | 2000 |
| C2 | Native 50, Recombinant 70 |
| C3 | 2000 |
| Objective | 100 |
|  | |
| **EPU parameters** | |
| Defocus range (-mm) | -2.0, -1.8, -1.6, -1.4, -1.2, -1.0, -0.8 |
| Autofocus frequency (mm) | Native 10, Recombinant 5 |
| Delay after stage shift (s) | 5 |
| Delay after image shift (s) | Native 3.5, Recombinant 1.5 |
| Exposures per hole | Native 1, Recombinant 3 |
| Grid tilt angle (°) | Native 0, Recombinant 20 |

## Table S7. Model refinement statistics

|  | Native | Recombinant |
| --- | --- | --- |
| Refined resolution (Å) | 2.31 | 2.25 |
| R-factor | 0.291 | 0.261 |
| Average Fourier shell correlation | 0.811 | 0.823 |
|  |  |  |
| R.m.s. deviations |  |  |
| Bond lengths (Å) | 0.013 | 0.010 |
| Bond angles (°) | 2.07 | 1.84 |
|  |  |  |
| Ramachandran plot |  |  |
| Favoured (%) | 95.5 | 97.0 |
| Outliers (%) | 0.6 | 0.4 |
|  |  |  |
| Mean B-factors (Å^2^) |  |  |
| Protein atoms | 29.1 | 28.5 |
| Waters | 12.9 | 14.4 |
|  |  |  |
| MolProbity clash score | 2.39 | 1.07 |
| MolProbity score | 1.88 | 1.46 |

**Supplementary methods**

## DDD02091966 synthesis and NMR

***N*-(3-(cyclopropylcarbamoyl)-4-fluorophenyl)-5-(phenoxymethyl)furan-2-carboxamide**

To a mixture of methyl 5-(phenoxymethyl)furan-2-carboxylate (70 mg, 0.30 mmol) and 5-amino-*N*-cyclopropyl-2-fluorobenzamide (70 mg, 0.36 mmol) in toluene (2 ml) was added trimethylaluminium (2 M in toluene, 450 μl, 0.90 mmol) drop-wise and the resulting mixture stirred at room temperature in a sealed tube for 18 hours. After cooling to 0°C, methanol (5 ml) was added drop-wise, the mixture concentrated *in vacuo* and crude product purified by flash chromatography (0 – 25% Cyclohexane / AcOEt:EtOH 3:1). Fractions were combined, triturated with methanol and dried to yield *N*-(3-(cyclopropylcarbamoyl)-4-fluorophenyl)-5-(phenoxymethyl)furan-2-carboxamide (60 mg, 0.15 mmol, 50 % yield)

**^1^H NMR (500 MHz, DMSO):** δ 0.51 – 0.56 (m, 2 H), 0.67 – 0.73 (m, 2 H), 2.84 (ddt, *J*=11.24, 7.52, 4.01 Hz, 1 H), 5.16 (s, 2 H), 6.81 (d, *J*=3.54 Hz, 1 H), 6.98 (t, *J*=7.12 Hz, 1 H), 7.06 (d, *J*=7.86 Hz, 2 H), 7.26 (t, *J*=9.32 Hz, 1 H), 7.29 – 7.38 (m, 3 H), 7.84 – 7.93 (m, 2 H), 8.37 (br d, *J*=3.54 Hz, 1 H), 10.34 (s, 1 H).

**HRMS** (ES^+^): *m*/*z* [M + H]^+^ calcd for C_22_H_20_N_2_O_4_F, 395.1407; found 395.1403.


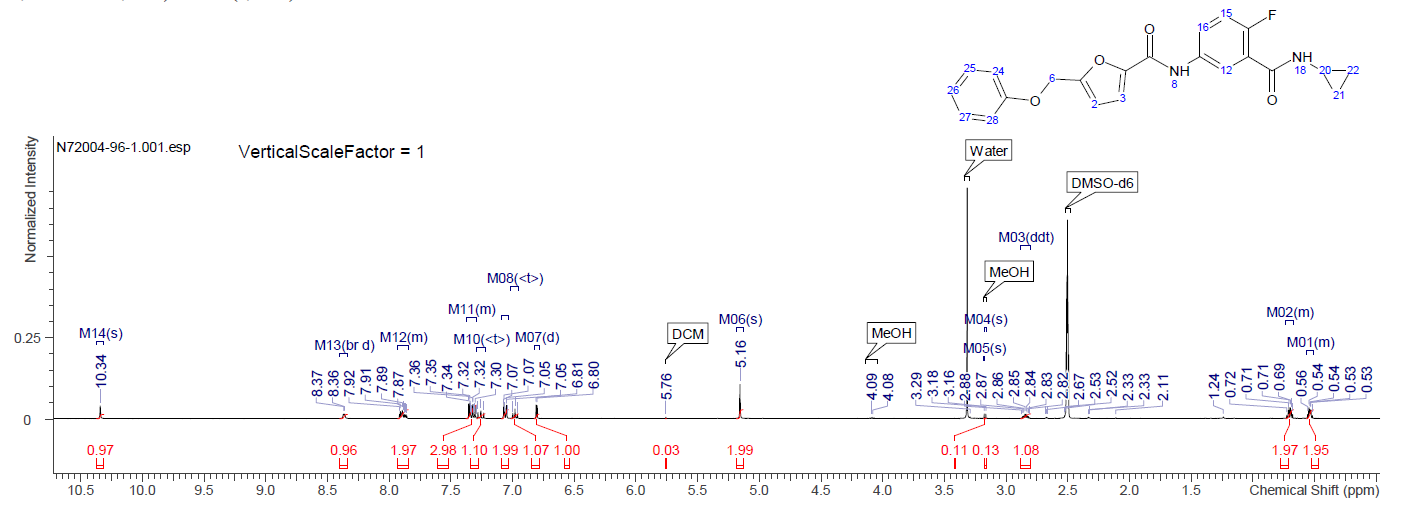


References

1 Waterhouse, A. M., Procter, J. B., Martin, D. M., Clamp, M. & Barton, G. J. Jalview Version 2--a multiple sequence alignment editor and analysis workbench. *Bioinformatics* **25**, 1189-1191, doi:10.1093/bioinformatics/btp033 (2009).

2 Wyllie, S. *et al.* Preclinical candidate for the treatment of visceral leishmaniasis that acts through proteasome inhibition. *Proc Natl Acad Sci U S A* **116**, 9318-9323, doi:10.1073/pnas.1820175116 (2019).

3 Thomas, M. *et al.* Scaffold-Hopping Strategy on a Series of Proteasome Inhibitors Led to a Preclinical Candidate for the Treatment of Visceral Leishmaniasis. *J Med Chem* **64**, 5905-5930, doi:10.1021/acs.jmedchem.1c00047 (2021).

4 Thomas, M. G. *et al.* Structure-Guided Design and Synthesis of a Pyridazinone Series of Trypanosoma cruzi Proteasome Inhibitors. *J Med Chem* **66**, 10413-10431, doi:10.1021/acs.jmedchem.3c00582 (2023).
